# In-Depth Understanding of the Effect of the Distribution of Substituents on the Morphology and Physical Properties of Ethylcellulose: Molecular Dynamics Simulations Insights

**DOI:** 10.1021/acs.biomac.4c00166

**Published:** 2024-06-24

**Authors:** Donghyun Kim, Patric Elf, Fritjof Nilsson, Mikael S. Hedenqvist, Anette Larsson

**Affiliations:** † Applied Chemistry, Department of Chemistry and Chemical Engineering, Chalmers University of Technology, SE-412 96 Gothenburg, Sweden; ‡ FibRe Centre for Lignocellulose-based Thermoplastics, Department of Chemistry and Chemical Engineering, Chalmers University of Technology, SE-412 96 Gothenburg, Sweden; § Department of Fibre and Polymer Technology, School of Engineering Sciences in Chemistry, Biotechnology and Health, KTH Royal Institute of Technology, SE-100 44 Stockholm, Sweden; ∥ FibRe Vinnova competence center, KTH Royal Institute of Technology, SE-100 44 Stockholm, Sweden; ⊥ Wallenberg Wood Science Center, KTH Royal Institute of Technology, SE-100 44 Stockholm, Sweden; # Wallenberg Wood Science Center, Chalmers University of Technology, SE-412 96 Gothenburg, Sweden; ∇ FSCN research centre, Mid Sweden University, 85170 Sundsvall, Sweden

## Abstract

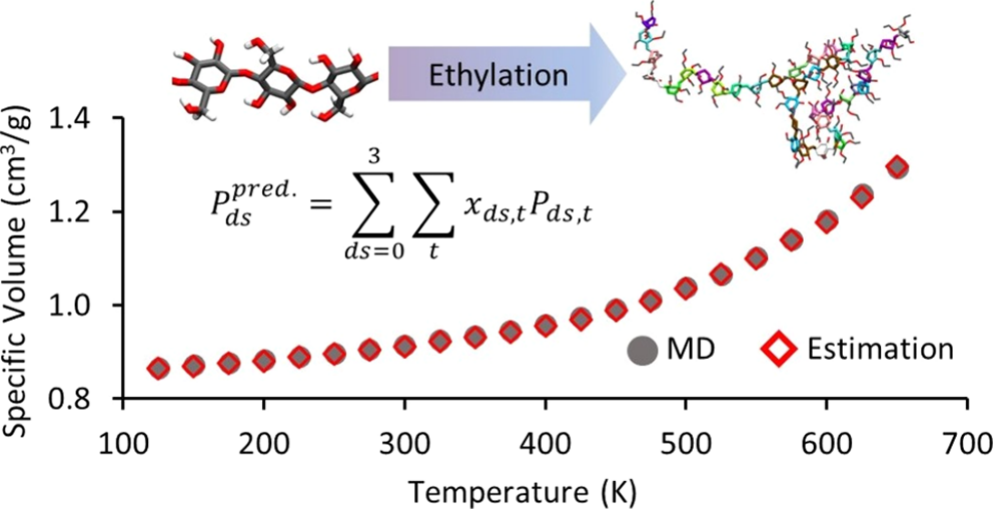

Ethylcellulose (EC) is a crucial cellulose derivative with widespread applications, particularly in the pharmaceutical industry, where precise property adjustments through chemical modification are imperative. The degree of substitution (DS) and the localization of substituents along the cellulose chains are pivotal factors in this process. However, the impact of the substituent location within the repeating unit of EC remains unexplored. To address this gap, we conducted molecular dynamics simulations on amorphous EC, comparing randomly and uniformly substituted ethyl groups in the repeating units. This comprehensive study of pairwise interactions revealed significant differences in intramolecular and intermolecular hydrogen-bonding capabilities, depending on whether the hydroxyl groups were substituted at C2, C3, or C6. While our simulations demonstrated that substituent localization in the repeating unit influenced the density, number of hydrogen bonds, and conformations, the DS emerged as the dominant determinant. This insight led us to propose and validate a hypothesis: a straightforward linear function using the properties of uniform models and molar fractions can predict the properties of randomly substituted EC with a given DS. This innovative approach is anticipated to contribute to the selection of cellulose derivatives with desirable properties for the pharmaceutical industry and new applications in other fields.

## 1 Introduction

Driven by mounting environmental concerns, substantial efforts have been directed toward replacing fossil-based plastics with biopolymers, which could be a more sustainable alternative.^1^ Since cellulose is one of the most abundant natural polymers, the use of cellulose could significantly contribute to a sustainable society by diminishing the consumption of fossil-based counterparts. Thanks to the characteristics of cellulose, such as biocompatibility, good mechanical properties, and biodegradability, it has been adopted in a wide range of applications, such as additives to foods, pharmaceuticals, ink, and building materials.^2^ To expand the usefulness of cellulose to most of the applications mentioned above, one should chemically modify the natural cellulose, leading to cellulose derivatives,^2^ such as cellulose ester and cellulose ether.

It is well-known that the design of the cellulose derivatives has a critical role in tuning their physical properties.^3,4^ Hence, applying various modifications to the cellulose backbone generates cellulose derivatives with a wide range of properties, e.g., from water-soluble to water-insoluble. This could open a huge area of possibilities to change the properties by, for example, selecting the type of substituent and the number of substituents per cellulose backbone. However, since different applications demand specific properties, one must actively choose which cellulose derivatives best meet the needs. To this end, one must understand how different substituents and the generated structures of cellulose derivatives affect their properties.

**Figure 1:**
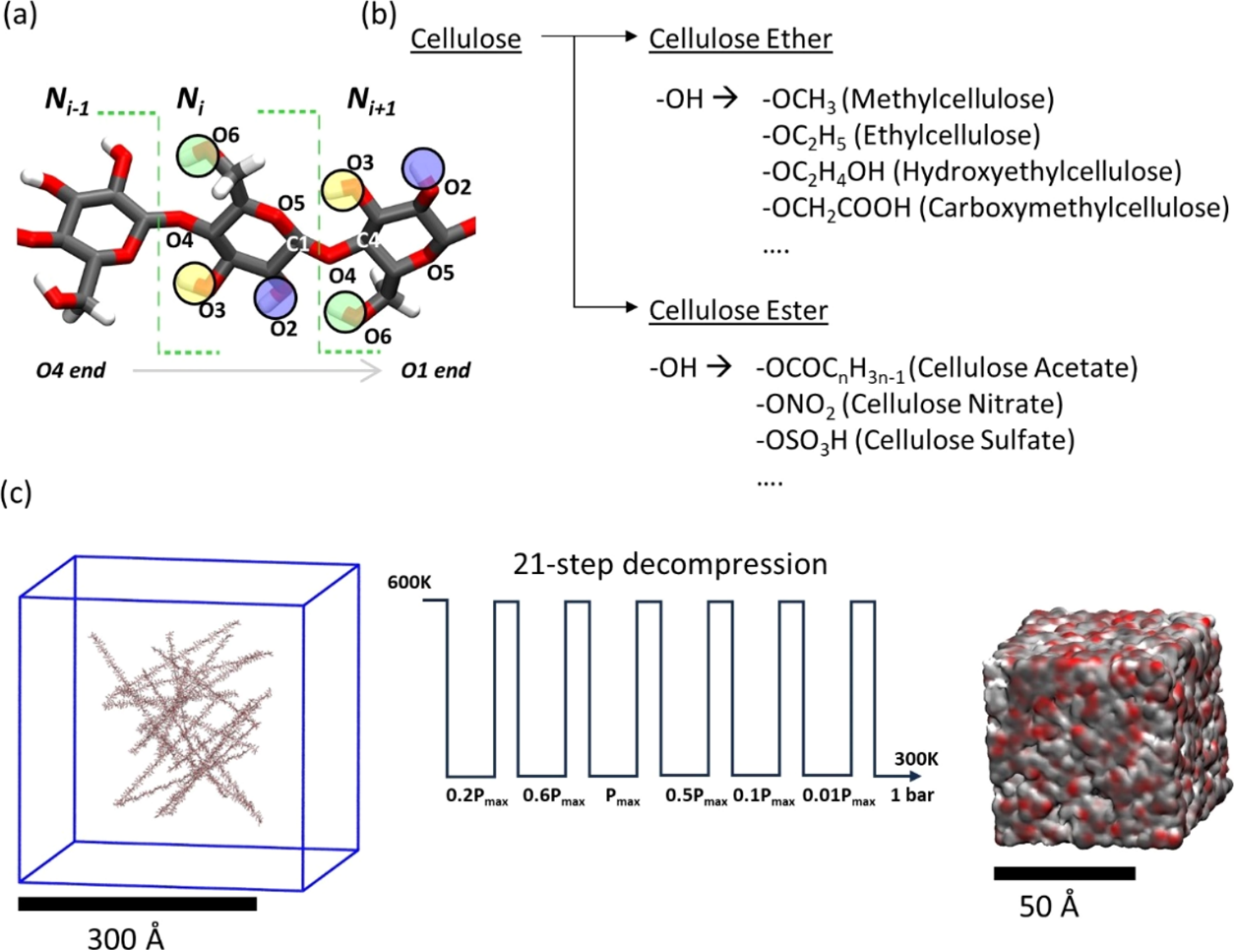
(a) Schematic illustration of a cellulose chain sequence where three hydroxyl groups at positions O2, O3, and O6 are subject to the substitution highlighted by the circles in purple, yellow, and lime colors, respectively. The green line denotes the borderline for a glucose unit. The number of glucose units increases from the O4 end (the nonreducing end) to the O1 end (the reducing end). (b) Examples of cellulose ethers and cellulose esters in the cellulose derivative family and (c) schematic picture of the 21-step decompression for U_ds3 model (fully substituted to DS = 3). The red, gray, and white colors in the simulation box correspond to oxygen, carbon, and hydrogen atoms, respectively.

Previous studies have also shown that not only the choice of substituents and degree of substitution (DS) but also the position of the substituents along the polymer chain and/or the position in the repeating unit (Figure 1a) could alter the properties.^3−6^ However, control over the design of the cellulose derivatives, particularly the position of the substituents, is not straightforward. In addition, various substituents lead to an enormous number of possible ways to chemically modify the cellulose chains. Hence, the degree of heterogeneity was used to study the effect of the distribution of substituents along the backbone. Larsson et al. used such an approach for hydroxypropyl methylcellulose (HPMC).^5,6^ In these studies, the increased heterogeneity of hydroxypropyl and methyl groups along the cellulose backbone decreases the release of drug from oral matrix tablets where, e.g., the release rate was shown to vary by a factor of 4, even if the DS was similar.

There exists a complex approach to exploring cellulose derivatives that are regioselectively (uniformly) substituted. Heinze and Emsley et al.^4^ have demonstrated the synthesis of 3-*O*-methylcellulose (DS = 0.99) and 2,3-*O*-methylcellulose (DS = 2.03) and characterized their structures by dynamic nuclear polarization magic angle spinning (DNP MAS) NMR, where they regioselectively methylated hydroxyl groups that were not protected by thexyldimethylsilyl (TDMS) followed by a deprotection step. Heinze et al. also synthesized 3-*O*-propyl cellulose with a range in DS from 0.19 to 1.02 via 2,6-di-*O*-thexyldimethylsilyl cellulose, characterized their structures, and studied the impact of DS and regioselectivity.^7^ It was shown that the cloud point of 3-*O*-propyl cellulose increases from 15.2 to 23.5 °C as the DS increases from 0.71 to 1.02. They also observed that the regioselectively substituted compounds were soluble in water, whereas randomly substituted compounds were insoluble. Other regioselectively modified cellulose include TEMPO-oxidized cellulose (at carbon C6) and dialcohol cellulose (with broken C2–C3 bonds).^8,9^

Ethylcellulose (EC) is a cellulose derivative that has been utilized in pharmaceutical formulations,^10,11^ heat-resistant chocolates,^12^ films,^13^ food packaging,^14^ ink formulations,^15,16^ etc., owing to its attributes such as being hydrophobic, light-stable, heat-resistant, and with the ability to stabilize dispersions. With these desirable properties, EC can be used as an excipient in various pharmaceutical formulations to enable production by, for example, being used as an additive in wet granulation liquids or to control the formulation properties, like masking the taste or controlling the drug release rate. Coatings comprising mixtures of EC with other cellulose derivatives, such as hydroxypropyl cellulose (HPC), have shown phase separation, leading to an improved, robust approach for tuning the drug release rate.^17,18^ The DS values for ECs intended for advanced applications are crucial, where ECs with low DS (0.5–1.5) are soluble in aqueous media, while ECs with DS in a range of 2.4–2.5 are soluble in both polar and nonpolar organic solvents and are often used to extend the drug release rate in controlled-release formulations.^19^ However, while the DS has been demonstrated to influence the properties of EC,^20^ there is limited knowledge regarding the impact of substitution patterns on its properties. Heinze et al.^21^ reported that the cloud point of 3-mono-*O*-EC, uniformly (regioselectively) substituted, is 30 °C higher than that of the randomly substituted EC. Huang et al. used molecular dynamic simulations of methylcellulose and showed that water interacts more with 3-mono-*O*-methylcellulose, where only the hydroxyl groups at carbon C3 were replaced with methyl groups, compared to when only C2 or C6 was substituted. It was also suggested that substitution of the hydroxyl group attached to C3 changes the conformation of the cellulose chain.^22^

In the pharmaceutical industry, precise control over the properties of the excipients and reliable estimation of the essential properties of each single batch are paramount. This requires well-characterized structures of the derivatives and access to established structure–property relationships. One approach to establishing such relationships for cellulose derivatives with different distributions of substituents could be via systematic syntheses and determinations of their properties, which are prohibitively challenging. Hence, molecular simulations, which can investigate the variation of cellulose derivative structures in detail, could be a useful tool for establishing such structure–property relationships.^23,24^

In this study, molecular dynamics (MD) simulations were used to explore how the physical properties of the EC are influenced by changes in DS and the locations of substituents in the repeating unit. To do this, MD simulations of amorphous EC with DS varying from 0 to 3 were performed, where the hydroxyl groups on the glucose units were either randomly substituted (random model) or regioselectively substituted (uniform model). Subsequently, the morphologies and properties of the resulting systems were investigated by estimating end-to-end distances (*R*_ee_), the radius of gyrations (*R*_g_), their pressure–volume–temperature (PVT) properties, the hydrogen bond (HB) densities, and intra- and intermolecular HB interactions between the different oxygens in the cellulose chains.

For amorphous EC, the HB formed between O3 and O5 is the dominant contributor to the intramolecular HBs, while for the intermolecular HBs, their strongest contribution comes from those with the participation of O6. For EC, the DS had a predominant impact on the conformations and PVT properties, whereas the distribution of substituents had a less impact on such properties. It was therefore hypothesized that it could be possible to predict several properties of EC with a given DS, by using a simple linear function of already known EC uniform models, including a pure cellulose system (i.e., DS = 0). To corroborate this idea, the properties of EC random models with DS = 1.5 and 2.5 were estimated using the simple linear function based on the results of simulations of the uniform models (DS = 0, 1, 2, and 3) and the estimates were compared to the results of MD simulations. The hypothesis was valid for properties such as the PVT curve, density, *R*_ee_, and *R*_g_. These results enhance our understanding of how EC properties are dependent on their molecular structures. Hopefully, this work can also serve as a guide for exploring other cellulose derivatives.

## 2 Methodology

### 2.1 Description of Systems

In this study, both random and uniform models were simulated to investigate the impact of the location of substituents, as well as the DS (see Table S1). To do this, a cellulose chain that consists of 36 repeating units was prepared using BIOVIA Discovery Studio Visualizer and 20 chains of it were randomly placed in an initial computational box using Packmol code^25^ (i.e., 36 × 20 = 720 anhydroglucose units in one system). Subsequently, the hydroxyl groups of each anhydroglucose unit at the carbon positions of C2, C3, and C6 were replaced with the ethyl groups to achieve the target DS and substitution type (i.e., either random or uniform model), as per Tables S1 and S2. The resulting EC systems were used for all-atom MD simulations.

The initial systems were equilibrated via a 21-step equilibration method.^26,27^ The systems were equilibrated in either isochoric–isothermal (NVT) or isothermal–isobaric (NPT) ensembles at several pressures (1–400 bar or 1–800 bar) and temperatures (300 or 600 K) (see Table S3) using the velocity rescale^28^ thermostat and Parrinello–Rahman^29,30^ barostat. In the ninth step of the 21-step decompression procedure, a maximum pressure of 800 bar was applied to the pure cellulose system (DS = 0), while 200 bar was applied to the other systems. A higher pressure was applied to the pure cellulose system to avoid occasionally occurring voids that complicate the analysis of the system. In the last step, step 21, the systems were equilibrated for 20 ns at 1 bar and 300 K in the NPT ensemble to yield the final system (Figure 1c). Figure S1a shows that the energy profile as a function of simulation time for the random model with DS = 2.5 (R_ds2.5) reached a plateau during the last step in the 21-step decompression, implying that the system is in an equilibrium state.

The EC systems resulting from the 21-step decompression were heated to 650 K and then cooled to 125 K to estimate the specific volume as a function of temperature. For each system, three samples with different initial configurations were simulated, and we assessed their properties averaged over three samples, such as density, HBs, *R*_ee_, and *R*_g_ after the 21-step decompression and during the cooling down process. The error bars in the figures discussed in the Results and Discussion section represent the standard deviations of the three samples.

### 2.2 Simulation Details

The GROMACS package (version 2021.3)^31,32^ was employed to carry out all-atom MD simulations of EC systems with the modified CHAMM36-jul2021 force field^33,34^ modified using the CGenFF server (see Table S4). For all MD simulations, the cutoff was set to 1.2 nm for both nonbonded interactions (van der Waals and Coulombic interactions) and the particle-mesh Ewald (PME)^35,36^ method was employed for long-range electrostatic interaction as implemented in the GROMACS package. All MD simulations were carried out with a 1.0 fs time step.

The HB density, HBs (number, length, and angle), *R*_ee_, and *R*_g_ of the systems resulting from the 21-step decompression were calculated by commands as implemented in the GROMACS package, which are *gmx energy*, *gmx hbond, gmx polystat, and gmx gyrate*, respectively. The dihedral angles (O5–C1–O4–C4 (called φ), C1–O4–C4–C5 (called ψ), O5–C5–C6–O6 (called ω), O2–C2–C3–O3, and O2–C2–C1–O4) in the chain were calculated using the MDAnalysis package.^37,38^

*R*_ee_ is defined as the distance between two hydrogen atoms of hydroxyl groups bonded to carbon 4 of the first monomer and carbon 1 of the last monomer in a chain. The *R*_g_ is defined as in eq 1
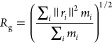
1where *m*_*i*_ and *r*_*i*_ are the mass of atom *i* and the position of atom *i* with respect to the center of mass of the molecules. In the cooling process, the systems were equilibrated at 1 bar at a given temperature (650–125 K at an interval of 25 K) in the NPT ensemble for 5 ns. At each temperature, the specific volume (=1/density) and HB density were determined. As illustrated in Figure S1b, the random model with DS = 2.5 (R_ds2.5) reached the convergence in terms of energy under the NPT run at 650 K. In addition, we carried out the simulations for longer equilibration time (10 ns), and the difference in the specific volume of the system between equilibration duration 10 and 5 ns appears negligible (less than 2%; see Figure S2). These results imply that a 5 ns equilibration at each given temperature during the cooling process is long enough.

## 3 Results and Discussion

### 3.1 Uniform Model: Density and HB Density

When it comes to modeling cellulose ethers, the most general assumption is that they are randomly substituted, making it challenging to study the effect of the location of substituents on their properties. Thus, we first simulated uniformly substituted EC systems (referred to as the uniform model) with DS = 0 (pure cellulose) and 1, 2, and 3 (fully substituted EC) to investigate how the location of each substituent affects the system. Figure S3 shows the snapshots of U_ds0 and U_O3 resulting from the 21-step decompression.

**Figure 2:**
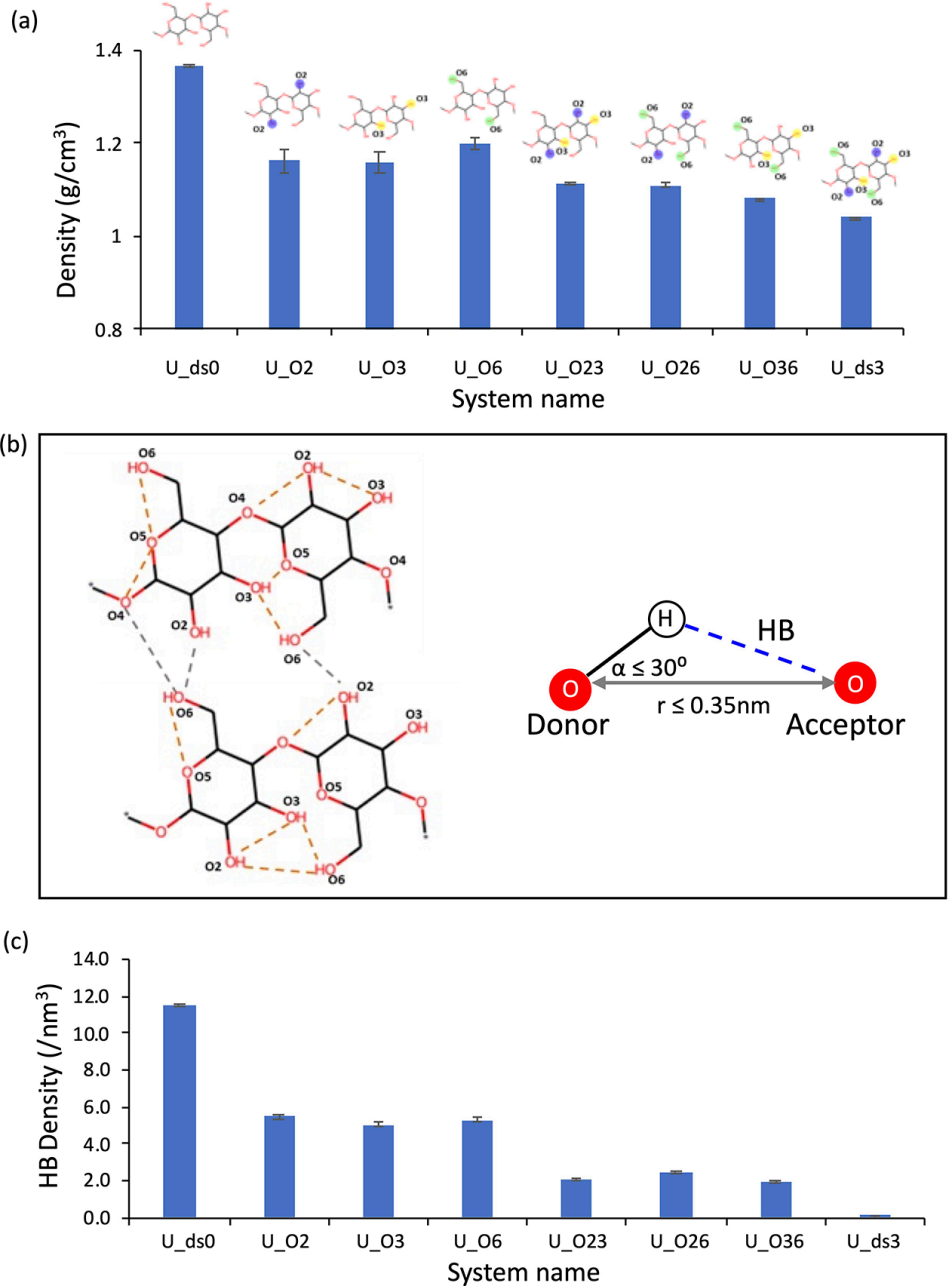
(a) Densities of the uniform models resulting from the 21-step decompression, where U in the system name stands for uniform distribution, ds0 and ds3 for non- and fully substituted ECs, respectively, and U_Oi and U_Oij correspond to EC with the ethyl groups substituted on carbons *i* and *j*, where *i* and *j* are = 2, 3, or 6. (b) A schematic illustration of HBs formed in cellulose chains where the intramolecular and intermolecular HBs are presented by dark yellow and gray lines, respectively, and (c) HB density of uniform models resulting from the 21-step decompression. All properties are the average of three samples, and the error bars are the standard deviation of the three samples for each property.

Figure 2a shows the density of the uniform models for different DSs (0, 1, 2, and 3), where the uniform models with DS = 1 and 2 contain three different systems, depending on where the substitution has occurred among the three hydroxyl groups attached to carbon 2, 3, or 6 in the repeating units; see Figure 1a and Table S1. Figure 2a shows that the density of EC decreased from 1.37 to 1.04 g/cm^3^ as DS increased from 0 (U_ds0) to 3 (U_ds3) (cf. 1.16–1.20 g/cm^3^ and 1.08–1.11 g/cm^3^ for DS = 1 (U_O2, O3, and O6) and 2 (U_O23, O26, and O36), respectively.), indicating an increase in the volume of the system with increasing DS due to the ethyl side groups placing the chains further apart.

The simulated density of pure amorphous cellulose (DS = 0) obtained from the present study was 1.37 g/cm^3^, which is comparable with previous computational (1.34–1.39 g/cm^3^) and experimental studies (1.48–1.50 g/cm^3^).^39−43^ Furthermore, the simulated systems of uniform models with DS = 2 had a slightly lower density (1.08–1.11 g/cm^3^) than the experimentally determined density of EC with DS = 2.1 (1.152 g/cm^3^) by Beck and Tomka.^20^ This decrease in the density of modeled systems compared to the experimental measurements for the amorphous systems of pure cellulose and EC could be attributed to the less ordered structure of the systems (i.e., less crystalline, semiordered, or paracrystalline region in the systems). Despite a slight difference in the density between the experiments and simulations, the trend of decreasing density with increasing DS, as observed in our simulations (Figure 2a), aligns with the experimental findings, where the densities of EC decreased from 1.48 to 1.114 g/cm^3^ over the DS ranging from 0 to 2.5.

In Figure 2b (left), a schematic illustration of all viable HBs in the system is displayed, where the number of HBs is counted when the distance between donor oxygen (OH) and acceptor oxygen (O) is less than or equal to 0.35 nm and the acute angle arising from a combination of the donor and acceptor is less than or equal to 30°, as shown in Figure 2b (right). Figure 2c shows that the HB density rapidly declined from 11.5 nm^–3^ (DS = 0) to 0.068 nm^–3^ (DS = 3) as the DS increased from 0 to 3. This result was associated with the decreasing number of hydrogen donor groups and the increase in the system′s volume (i.e., decreasing density, as shown in Figure 2a) with the DS. The occurrence of hydrogen bonding (HB) in the U_ds3 system is due to the presence of one −OH group in each repeating unit at the ends of the chains, which is not substituted and can act as a donor.

**Figure 3:**
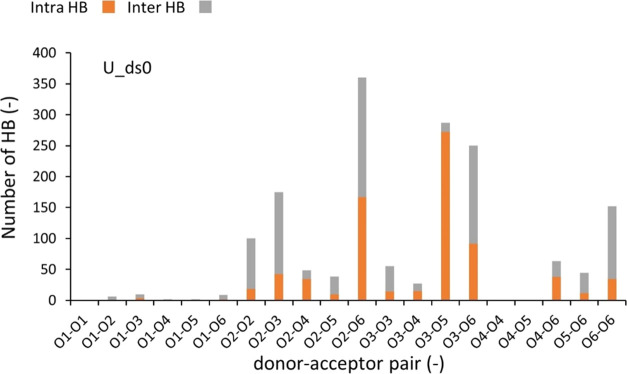
HB with respect to the donor–acceptor pair for U_ds0 (pure cellulose system), with the number of intramolecular HBs (orange) and intermolecular HBs (gray).

In addition, the ability of the different oxygen atoms to form HB is described in Table S5 and the HB distributions of uniform models with respect to donor and acceptor pairs (i.e., pairwise HB distribution) were investigated to understand how the location of the substituent affects the formation of HBs in the EC systems. Figure 3 shows a pairwise HB distribution of the amorphous system of pure cellulose (U_ds0). The major contributors to the total HBs of pure cellulose were the pairs of compounds O2–O2, O2–O3, and O2–O6, and pairs of compounds O3–O5, and compounds O3–O6 and O6–O6 pairs. This result makes sense since all hydroxyl groups at the carbon 2, 3, and 6 positions are unsubstituted and can contribute to forming HBs.

To gain deeper insights into the formation of HBs for amorphous cellulose, we differentiated the intramolecular HBs (Figure 3, orange) and the intermolecular HBs (Figure 3, gray). The number of intermolecular HBs exceeded intramolecular HBs for the O2–O2, O2–O3, O3–O6, and O6–O6 pairs. On the other hand, the intramolecular HBs for the O3–O5 pair accounted for a much larger portion of total HBs than its intermolecular HBs. These HB results agree with an earlier MD study on amorphous pure cellulose.^39^

**Figure 4:**
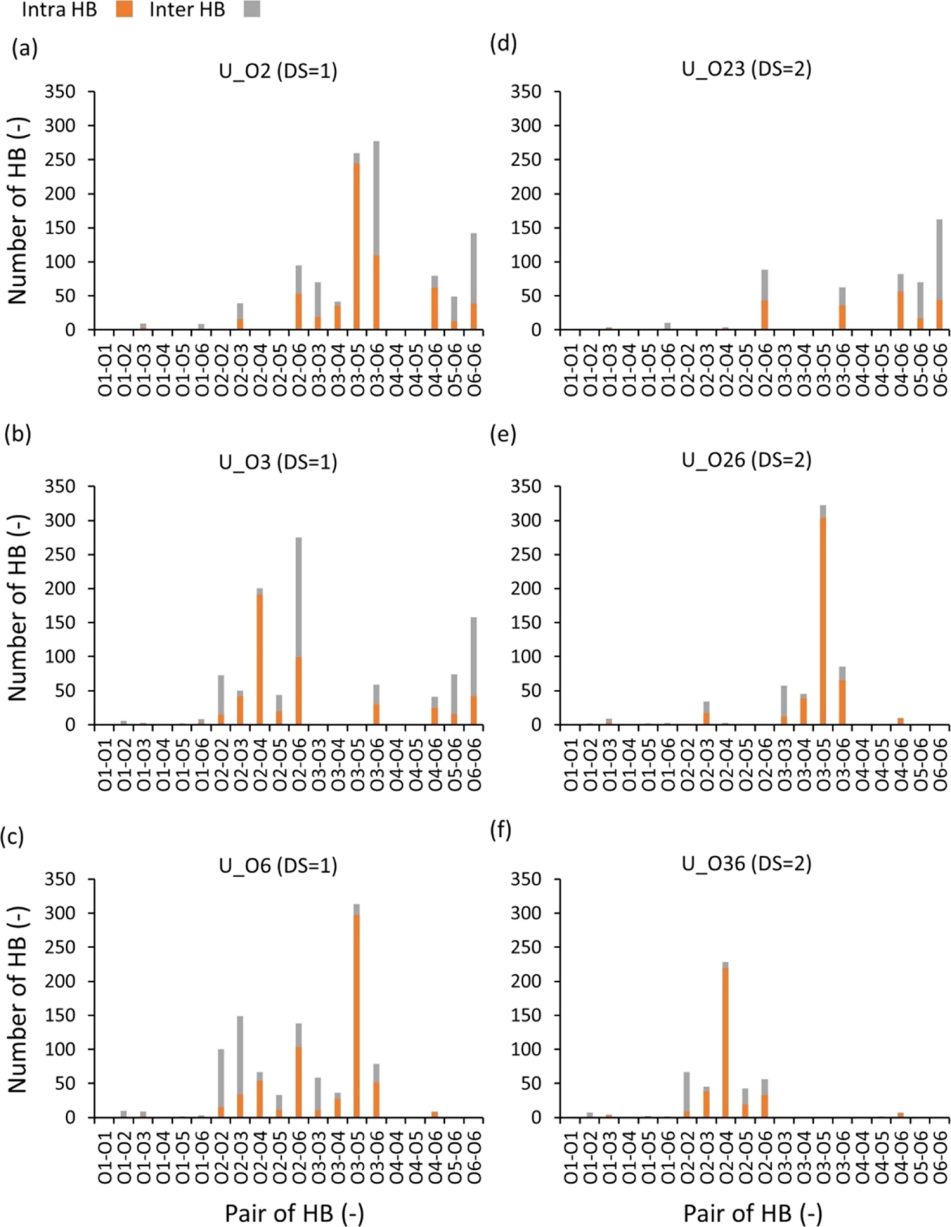
Pairwise HB distribution for uniform models with the DS = 1 (a–c) and 2 (d–f). The number of intramolecular and intermolecular HBs is presented in orange and gray columns, respectively.

**Figure 5:**
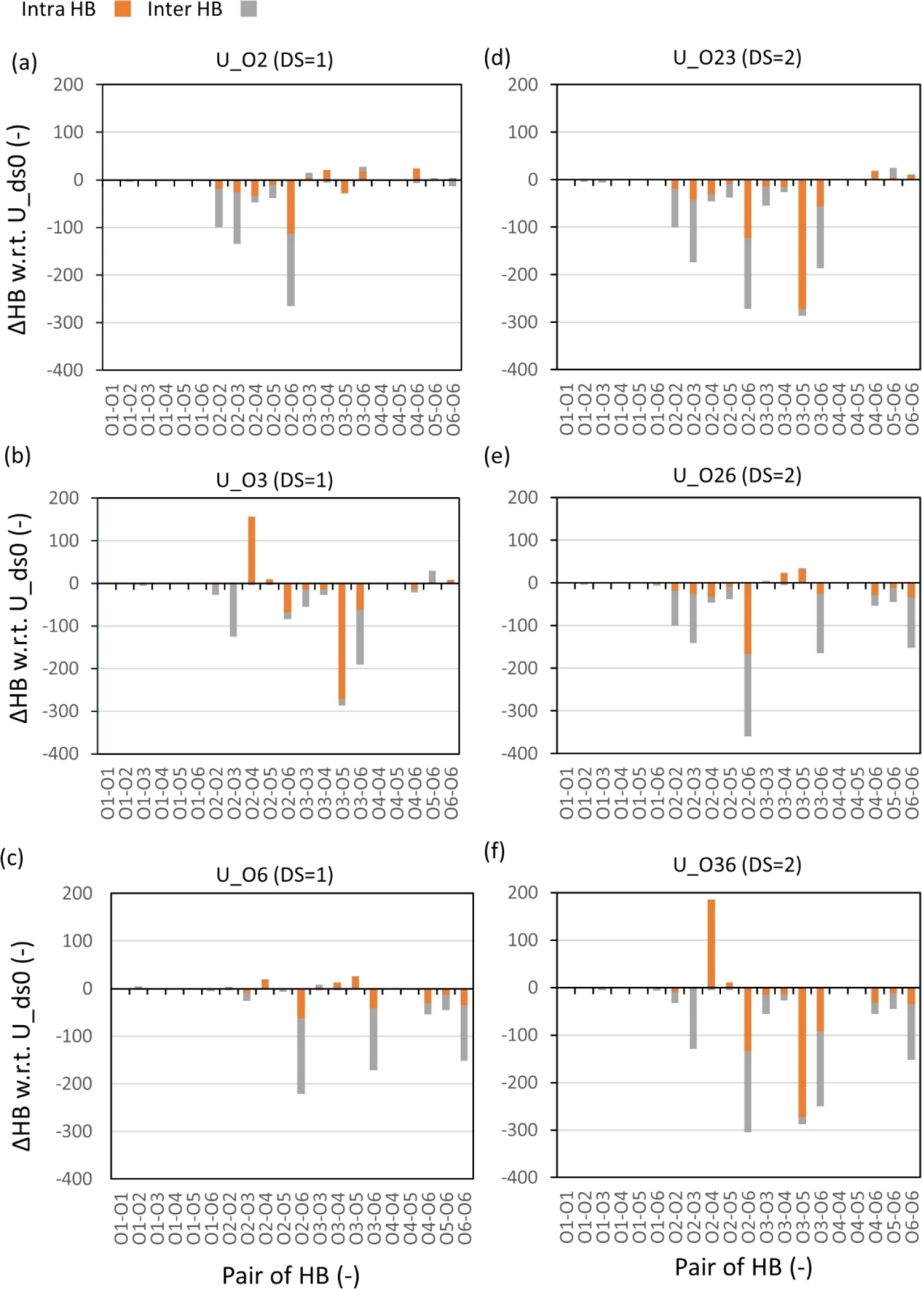
Change in the number of pairwise HB (ΔHB = *N*_uniform_ – *N*_U_ds0_) when the chains are uniformly substituted to DS = 1 (a–c) and 2 (d–f), where orange and gray colors denote the intramolecular and intermolecular HBs, respectively.

The pairwise HBs for the uniform models with DS = 1 and 2 were also analyzed, as shown in Figure 4. For three uniform models with DS = 1 (with ethyl groups at the hydroxyl groups attached to carbons 2, 3, and 6, corresponding to the system names U_O2, U_O3, and U_O6, respectively), the number of HBs decreased significantly compared to pure cellulose (see Figures 3 and 4a–c). Furthermore, a distinct difference in the pairwise HB distributions among the three uniform models with DS = 1 was observed (Figure 4a–c). Figure 5a–c shows the change in the pairwise HB of given uniform models with DS = 1 relative to the unsubstituted model. Such a decrease in the number of HB induced by substitution could be due to several factors, such as a decrease in the number of donor groups, steric hindrance inducing conformational changes in the chain, reducing the chance of forming HB by breaking the contact between the donor and acceptor, and so on.

Starting with comparisons of pairwise HB within the chains (i.e., intramolecular HB, orange bars in Figures 3–5), it becomes evident that the number of intramolecular HBs in unsubstituted EC (pure cellulose, U_ds0) was the greatest between O3 and O5 (Figure 3), followed by the O2–O6 pair. Remarkably, this was also the case for U_O2 and U_O6, where the hydroxy groups at carbon 3 (O3) remain unsubstituted. In fact, the number of intramolecular O3–O5 HB interactions was seldom affected and close to the nonsubstituted cellulose chains (native), indicating that the HBs within the EC chains are not disturbed if the O3 is not substituted. However, when O3 is substituted (U_O3), the O2–O4 pairs become the dominant intramolecular HB in the system (Figures 4b and 5b), which is explained by the lack of a donor in the O3–O5 pair. Table S5 shows whether an oxygen position is a donor or acceptor for a given substitution.

**Figure 6:**
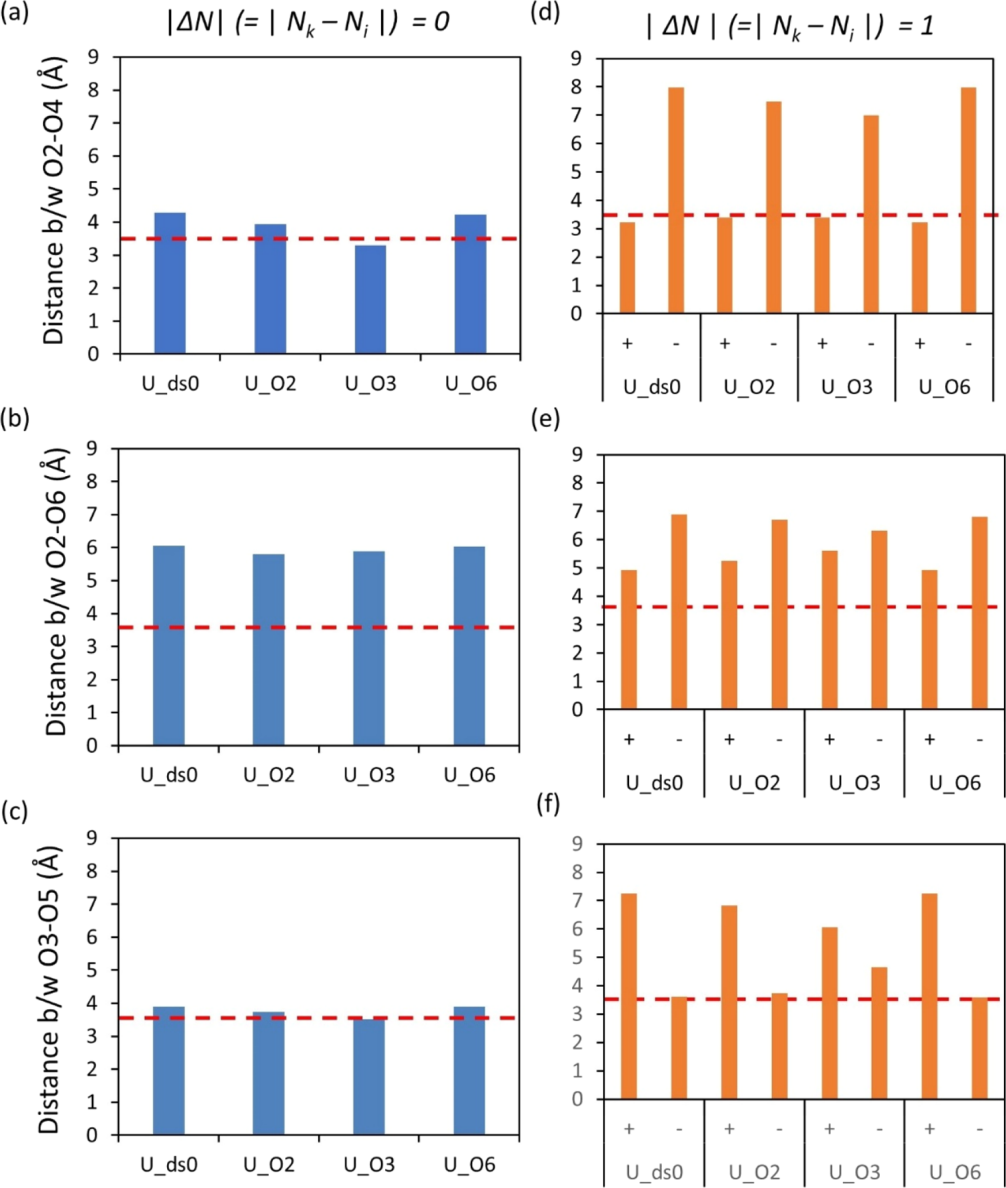
Average distances are displayed between two oxygen atoms in three HB pairs (O2–O4, O2–O6, and O3–O5) within one unit (a–c), whereas in panels (d–f), it is between two nearby units. The + and – denote the direction of the interactions (O4 end → O1 end) and (O1 end → O4 end), respectively. The red horizontal line is the cutoff bond length for HB. Note that these results do not show the bond length of HB.

Surprisingly, in U_O3, a significantly larger number of O2–O4 HB interactions were found than in U_ds0 (Figure 5), whereas the opposite was found for the U_O2 and U_O6 models. This observation suggests that substituting the hydroxyl groups at O3 would impose steric hindrance on the chains, which could change the conformation of the chains as shown in Figures 6 (the distance between the donor and acceptor in the chain. Note that this is not the length of HB) and 7 (the dihedral angles φ,ψ (space), O2–C2–C3–O3, and O2–C2–C1–O4). Figure 6a shows the distance of the O2–O4 of the U_O3 model (O3 substituted) within the same glucose unit (|Δ*N*| = 0; Figure 6a). A subtle shortening of the distance between O2 and O4 for U_O3 compared to that for U_ds0 was observed, whereas no clear change relative to U_ds0 could be observed when O2 (U_O2) or O6 (U_O6) is substituted.

**Figure 7:**
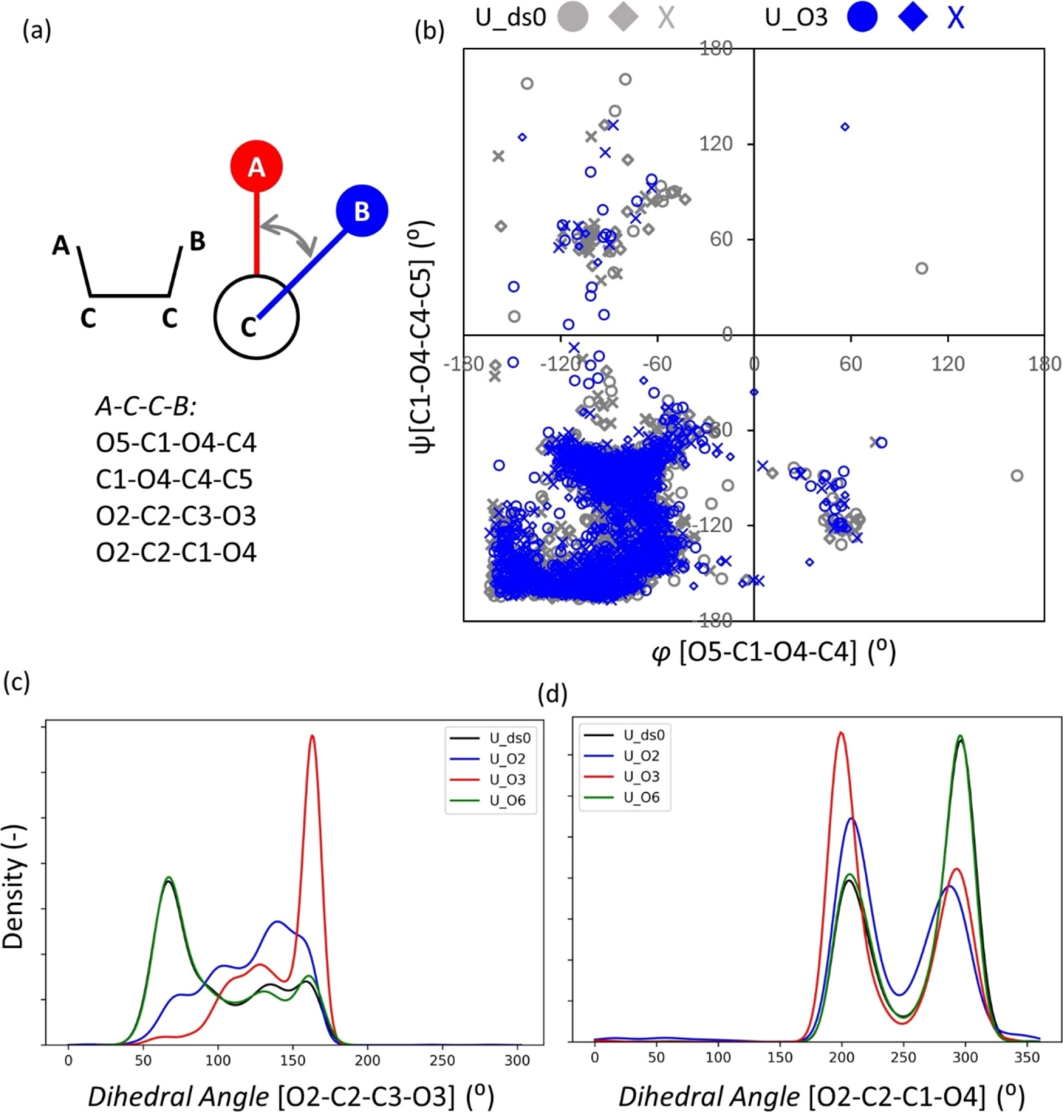
(a) A conceptual scheme of dihedral angles φ and ψ in the chain and the distribution of dihedral angles for U_ds0 and U_O3, (b) [φ, ψ] plot, (c) O2–C2–C3–O3, and (d) O2–C2–C1–O4. The marks of circle, rhombus, and *x* denote three replicate simulations.

As illustrated in Figure 7a, we also analyzed the dihedral angles around the β-1,4-glycosidic linkage ([φ, ψ]; Figure 7b) and the C2 position in the glucose unit (Figure 7c,d) as well as the O6 position (ω; Figure S4a,b). Figure 7b demonstrates that the distribution of [φ, ψ] for amorphous U_ds0 exhibits a cluster close to its average (−87°, −125°), which is a typical configuration of the β-1,4-glycosidic linkage and is in good agreement with previous MD studies.^22,39^ The distribution of [φ, ψ] for U_O3 appears similar to that of U_ds0, and also U_ds3 is similar to U_ds0, as shown in Figure S4. On the other hand, the dihedral angles including O2–C2 (Figure 7c,d) change significantly when O3 is fully substituted:

(1) O2–C2–C3–O3: U_ds0 shows a main peak at around 68° (Figure 7c, black line) and after then a broad peak until around 170°, while U_O3 has a big and narrow peak at around 165° (Figure 7c, red line), indicating that the OH groups at the C2 position of U_O3 prefer to stick out in the opposite direction from the OH group at the C3 position. These changes in the conformations alter the HB bond distance (Figure 6a) and dihedral angle (Figure 7c) and could be associated with the formation of intramolecular HB between O2–O4 within the same glucose unit (Figure S5b), whereas we can see the highest intramolecular HB for U_ds0 between two neighboring glucose units (|Δ*N*| = 1 in Figure S5a).
(2) In U_ds0, there are two main peaks around 205 and 305° (Figure 7d, black line) for the O2–C2–C1–O4 dihedral angle. When O3 is substituted, the peak around 205° increases, while the one around 305° decreases (Figure 7d, red line). This suggests that more OH groups at C2 rotate anticlockwise relative to O4 in the next glucose unit (*N*_*i+1*_). These results explain why we see more intramolecular HB between O2 and O4 within the same glucose unit in U_O3 (Δ*N* = 0), rather than between two adjacent glucose units. Despite the shorter distance between O2 and O4 in two adjacent glucose units (Figure 6d), the preference for intramolecular HB within the same glucose unit (Δ*N* = 0) in U_O3 seems counterintuitive based on the depicted sketch of the initial conformation of pure cellulose chains (see Figure 1a). This suggests that the change in conformation caused by the substitution of O3 with the ethyl group plays a crucial role in intramolecular HB formation.

In addition, we can also see an evolution of the peak corresponding to the O5–C5–C6–O6 dihedral angle (ω) at about 142° for U_O3 as shown in Figure S4a (red line), while U_ds0 has a broad distribution in a wide range from 0 to 200° and a smaller peak at about 300° (Figure S4a, black line). This indicates that U_O3 prefers to have a Trans–Gauche (TG) orientation, while amorphous pure cellulose (U_ds0) can take all conformations (Gauche–Trans (GT), Trans–Gauche (TG), and Gauche–Gauche (GG)). On the other hand, other uniform models with DS = 1 (U_O2 and U_O6) exhibit only moderate change in their chain conformations in terms of the dihedral angles compared to U_O3 (Figures 7c,d and S4a).

For the systems in which O3 is not substituted (i.e., U_ds0, U_O2, and U_O6), the distance between O3 and O5, both in the same glucose unit (Δ*N* = 0, Figure 6c) and in two adjacent glucose units (Δ*N* = 1, Figure 6f), is relatively short compared to O2–O6 (Figure 6b,e). Furthermore, the dihedral angle of the two-cation-two-component complex of O2–C2–C3–O3 is larger for U_O3 than for the other models (Figure 7c). These changes in the conformation of chains by the substitution of the O3 substituent corroborate that the highest peak of intramolecular HB is the O3–O5 substituent for the systems where the O3 substituent is not substituted (Figures 4 and 5). As Figures 4, 5, S6a, and S15a indicate, despite a substantial increase in intramolecular HBs between O2 and O4, the total number of intramolecular HB for U_O3 is still lower than that of U_O2 and U_O6. From these results, we conclude that whether or not O3 was substituted has a more important role in forming intramolecular HBs.

Analysis of the intermolecular HBs of unsubstituted EC (pure cellulose, U_ds0) showed that donor–acceptor pairs involving O6, such as those involving O2–O6, O3–O6, and O6–O6, had a higher number of HBs than other pairs (Figure 3). This observation also applies to EC systems with DS = 1 having unsubstituted O6 (U_O2 and U_O3, Figure 4b,c). Figure S6b shows that the intermolecular HB density for unsubstituted EC chains was more than twice that of all EC chains with DS = 1. From this finding, we can infer that the ethyl side groups placing the chains apart increase the volume of the system and decrease the intermolecular HB density, resulting in a weaker interchain interaction.

The pairwise HB distributions for the uniform models with DS = 2 (with ethyl groups at the hydroxyl groups attached to carbon 2,3 or 2,6 or 3,6, denoted as U_O23, U_O26, or U_O36, respectively) are shown in Figures 4d–f and 5d–f. When the hydroxyl groups at the carbon 2 (O2) and 3 (O3) positions are fully substituted (U_O23), no pronounced intramolecular HB peaks appear. This is because the donor groups (−OH at the O2 and O3 positions) for the most prominent intramolecular HB pairs observed for DS = 1 (O2–O4 and O3–O5; see Figure 4a–c) are replaced with the ethyl groups. Interestingly, a pronounced intramolecular HB peak for U_O26 was observed for the O3–O5 pair, with a similar number of intramolecular O3–O5 HBs as for the unsubstituted EC (DS = 0; Figure 5). This emphasizes the fact that when O3 is not substituted, the main contributor to the number of intramolecular HBs is unaffected, as described in the analysis of uniform models with a DS = 1 above. Uniform models with DS = 2 demonstrate similar features as the uniform models with DS = 1, in terms of the distribution of the number of HB as a function of HB length in the unit of the index of glucose (Figures 1a (index) and S5c (distribution)) and dihedral angles (Figures S4b and 7a,b).

Based on the pairwise HB distribution analysis for uniform models (Figures 3–5), we could infer that the positions of O3 and O6 play crucial roles in forming intramolecular and intermolecular HBs, respectively. Such roles of the O3 and O6 positions in intra- and intermolecular HB formations, respectively, were attributed to the proximity of the hydroxyl group at the O3 to the O5 position in the repeating glucose units along the EC backbone and the outward orientation of the hydroxyl groups from the carbon 6 position (O6). Figure S6a shows that a decrease in the intramolecular HB density induced by substitution at the O3 position (U_O23 vs U_O2 and U_36 vs U_O6) was greater than that by substitution at the O2 or the O6 position (U_O26 vs U_O2 or U_O6), corroborating the role of the O3 in forming intramolecular HBs. In addition, a decrease in the intermolecular HBs induced by substitution at O6 (U_O26 vs U_O2 and U_O36 vs U_O3) was greater than by substitution at the O2 or O3 positions (U_O23 vs U_O2 or U_O3), corroborating the role of O6 in forming intermolecular HBs.

The literature^44^ shows that the hydroxyl group at O3 is less preferred to become substituted,^45−47^ which has been traditionally explained by lower accessibility of the reactants. This behavior of pairwise HB distribution depending on the location of substituents allows us to expect that the properties of the EC system will vary depending on the location of substituents as well as the DS. This idea will be discussed together with the random models presented in the following sections.

### 3.2 Random Model

In Section 3.1, the uniformly substituted EC models were discussed, representing a more straightforward system and revealing the effects of specific positions of the substituents in the repeating units more easily. However, as described before, cellulose ethers are generally randomly substituted. Therefore, this section presents the results of randomly substituted EC systems with DS varying from 0 to 3. In these models, the hydroxyl groups are randomly substituted with ethyl groups in the repeating glucose units and across the chains. The results from the random models are compared to the uniform models discussed in Section 3.1.

#### 3.2.1 Structure and HB Density of the Random Model

**Figure 8:**
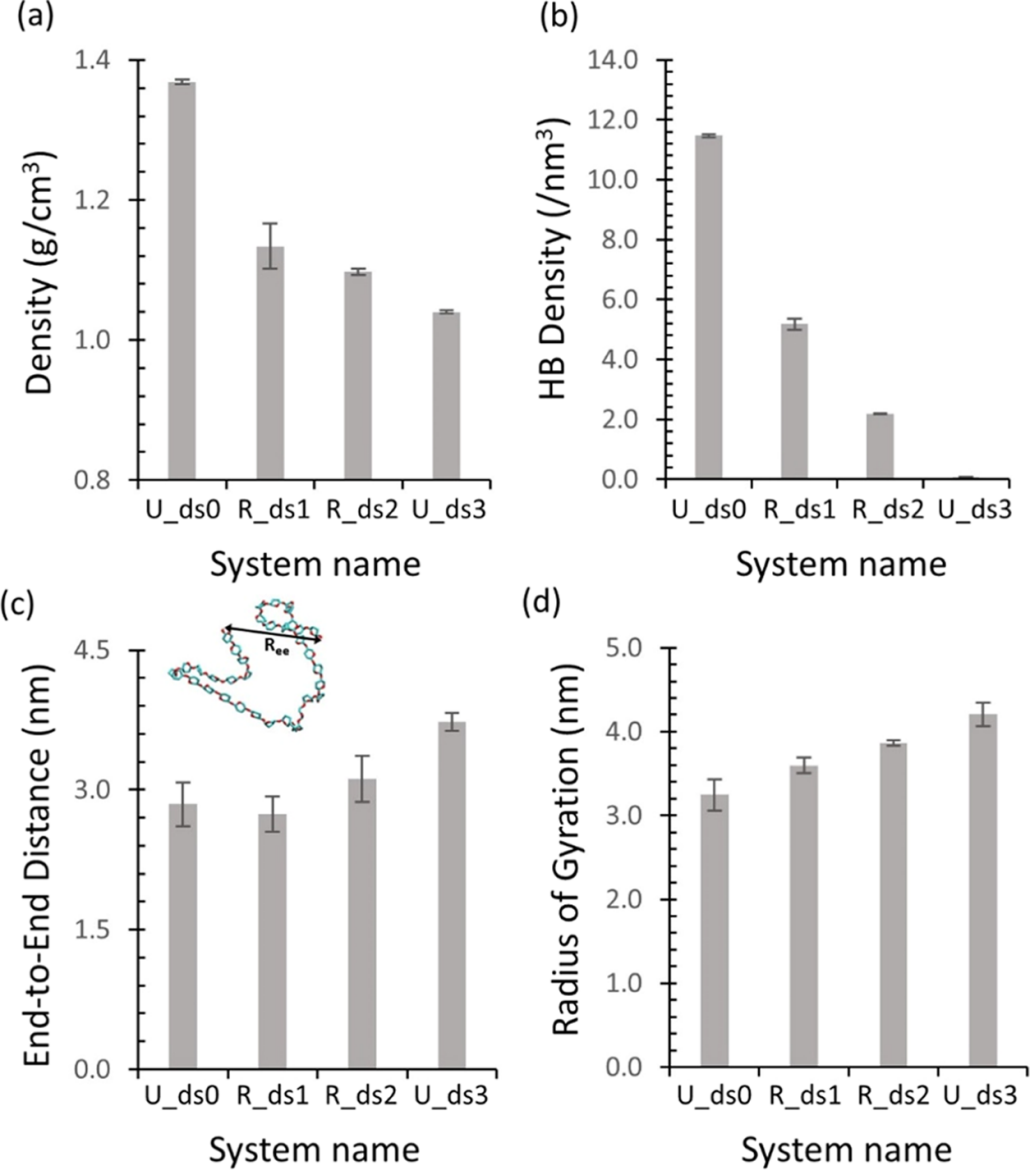
(a) Density, (b) HB density, (c) *R*_ee_, and (d) *R*_g_ of random models (U_ds0, R_ds1, R_ds2, and U_ds3). Note that U_ds0 and U_ds3 represent uniform systems.

Figure 8 presents the density (Figure 8a), (total) HB density (Figure 8b), *R*_ee_ (Figure 8c), and *R*_g_ (Figure 8d) for randomly substituted systems with a DS = 0, 1, 2, and 3 (referred to as U_ds0, R_ds1, R_ds2, and U_ds3, respectively; see Table S1). The random models with DS = 0 (U_ds0) and 3 (U_ds3) are the same systems as the uniform models with DS = 0 and 3 discussed in Section 3.1. Similar to the uniform models (Figure 2a,c), the random models show a gradual decrease in both density and HB density with increasing DS (Figure 8a,b). Figure S6b shows that both intramolecular and intermolecular HB densities of systems decreased with increasing DS. Notably, the intermolecular HB density for unsubstituted cellulose (U_ds0; see Figure S6b, gray bar) was larger than its intramolecular HB density (orange bar, Figure S6b). However, when the DS ≥ 1, the HB densities become reversed (i.e., the intermolecular HB densities were smaller than the intramolecular HB); see Figure S6b. This, together with the decreased total HB densities with increased DS, may be one factor that contributes and partially explains why the glass-transition temperature (*T*_g_) decreases with increasing DS.^20^Figure S8 shows the average angle and bond length of HB for both uniform and random models, where the average angle is smaller than 25°, and the average HB length is shorter than 3.0 Å for all models, and there is a subtle difference among models.

The decrease in the density with increasing DS was attributed to loosely packed chain arrangements induced by bulky ethyl groups. The replacement of hydroxyl groups with ethyl groups reduces the number of donor–acceptor pairs that can form HBs, and the bulky ethyl group can also increase the distance between the donor–acceptor groups and change the conformation of the chains, resulting in a decrease in the number of HBs. These results indicate that the chains would undergo less contraction and adopt a more expanded conformation as the DS increases due to less intramolecular interaction and the steric hindrance of the ethyl groups. This idea can be corroborated by increasing *R*_ee_ and *R*_g_ with the DS as illustrated in Figure 8c,d. The changes in *R*_ee_ and *R*_g_ with DS imply that the chains contract or aggregate less due to substitution with ethyl groups, which introduces changes in the chain conformations. The decreased *T*_g_ also suggests that the chains become more flexible with increasing DS. A recent publication demonstrated that breaking the covalent bond between carbons 2 and 3 of glucose units, resulting in dialcohol cellulose, enhances the chain’s flexibility and that the density decreased with increasing the number of broken C2–C3 bonds, which is consistent with our results.^43^

#### 3.2.2 Pressure–Volume–Temperature (PVT) Property of Random Models

**Figure 9:**
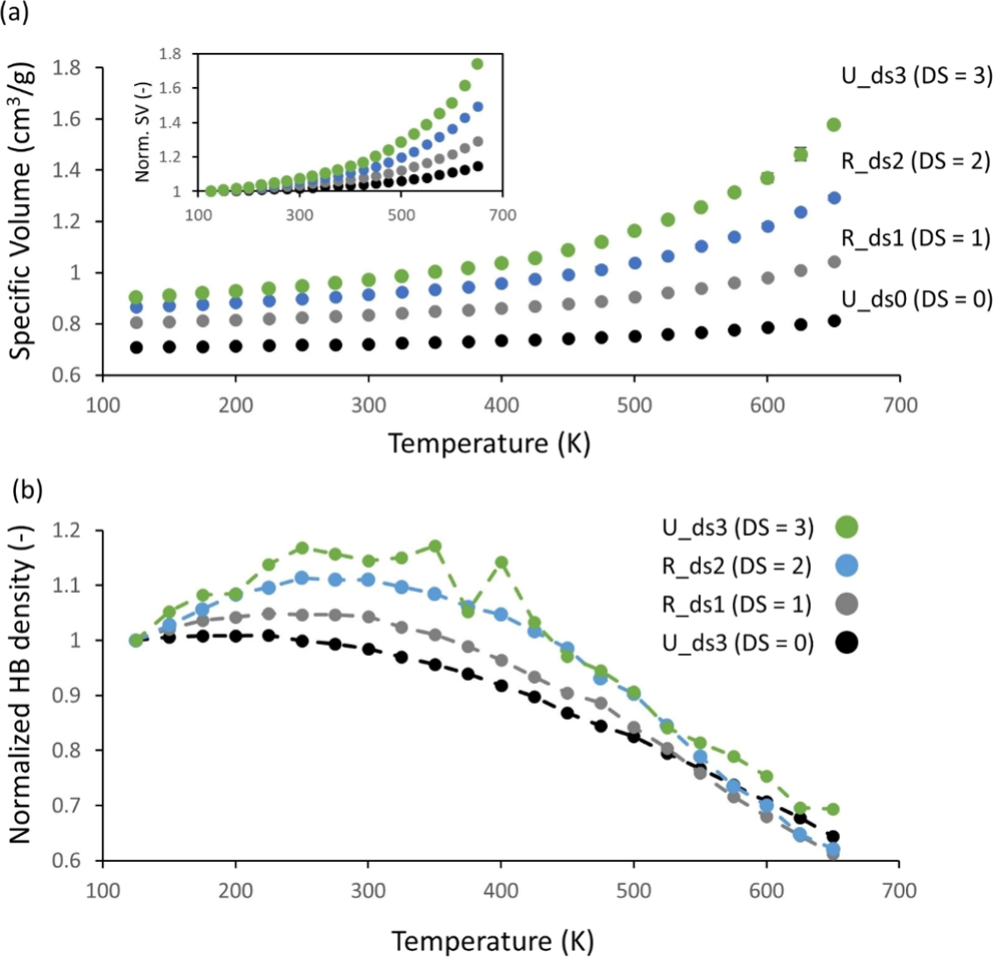
(a) Pressure–volume–temperature curves of random models (U_ds0, R_ds1, R_ds2, and U_ds3), where the inset is the PVT curves normalized with the specific volume at 125 K, (b) a change in the HB density of U_ds0, R_ds1, R_ds2, and U_ds3 models during the cooling down process.

Figure 9a shows the specific volume (*v*_sp_ = 1/density) during cooling from 650 to 125 K, sampled at an interval of 25 K. In general, a bilinear PVT curve is expected, where the slope changes clearly at (or near) the *T*_g_. From this perspective, *T*_g_ could be estimated by finding an intersection point, i.e., the crossing point of two lines in the bilinear PVT curve. However, it is not straightforward since the resulting PVT curves in the present study exhibited a hyperbolic shape rather than a bilinear one. This induces non-negligible uncertainties using the traditional approach (i.e., bilinear fitting) to determine *T*_g_, as it becomes dependent on several factors, such as the range of temperature to fit the lines and the upper limit temperature of the cooling simulation, making the determination of *T*_g_ more complex.

Despite such uncertainties in determining *T*_g_ from the PVT curve, the effect of the DS on *T*_g_ can still be qualitatively explored by comparing the PVT curve of random models for the investigated DS values. As shown in Figure 9a, random models with a higher DS showed a faster change in the specific volume with temperature, implying that *T*_g_ gradually decreases with increasing DS from 0 to 3, which the experiments have confirmed. This qualitative comparison could be made more clearly by normalizing the PVT curves with their *v*_sp_ at 125 K, as shown in the inset of Figure 9a. Such a decrease in *T*_g_ with increasing DS was attributed to the less packed system and higher chain mobility induced by the decreasing HB density with the DS due to bulky ethyl groups. In Table S6, we show *T*_g_ estimated by finding the temperature from which the specific density deviates more than 1% from a linear fit to six points at low temperature (125–250 K). This estimation also corroborates that the qualitative comparison of *T*_g_ above that decreases *T*_g_ with DS. Interestingly, even if there are uncertainties in determining the *T*_g_ by MD simulations, the simulated *T*_g_ of random model with DS = 2.5 is 325–350 K, and it is comparable with the experimental data to be around 400 K.^48^ The difference between the experiments and simulations suggests that the simulations should be used for comparison and to identify trends.

**Figure 10:**
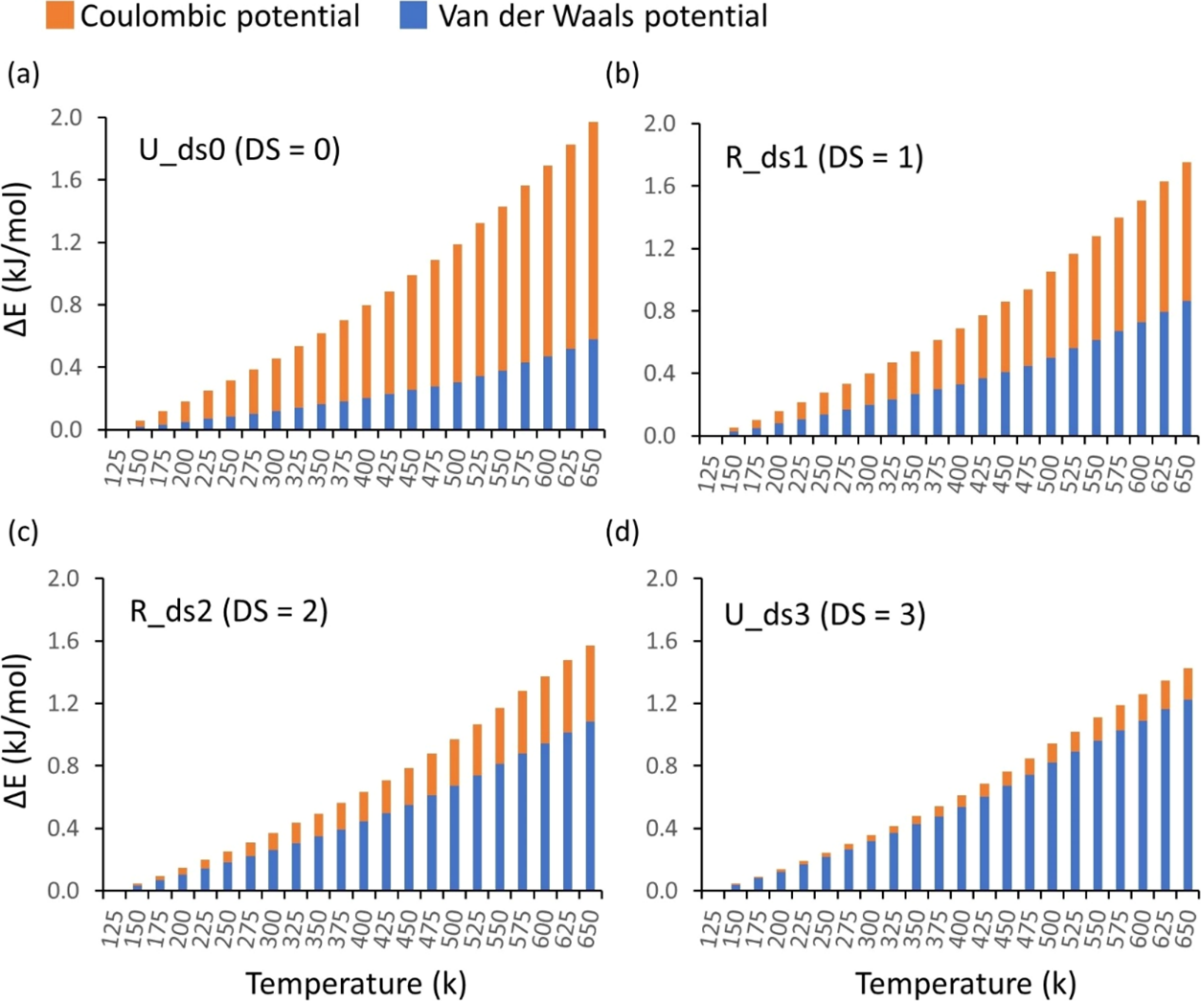
A change in the nonbonded potential energy that consists of the Coulombic potential (orange bar) and van der Waals potential (blue bar) approximated by the Lennard-Jones potential with respect to temperature in the systems for a DS = 0 (a), 1 (b), 2 (c), and 3 (d). The energy is normalized to the total number of atoms.

Subsequently, the change in HB density during the cooling process was analyzed to understand the PVT behavior further. Figure 10b shows HB density as a function of temperature normalized with the value at 125 K. All systems with a DS from 0 to 3 (U_ds0, R_ds1, R_ds2, and U_ds3) demonstrated that the HB density increases with an increasing temperature between 125 and 225–350 K and then decreases with a further increase in temperature. This means the number of HBs increases at low temperatures (125–350 K), while the system expands as the temperature increases. The system with a DS = 3 shows a volatile behavior compared to other systems, which was attributed to its low HB density (see Figure 8b). This result infers that at low temperatures (125–350 K), the increasing temperature induces more mobility to the polymer chains and more space due to the expansion of the system, enabling the formation of HBs.

On the other hand, polymer chains have higher energies and larger mobility at high temperatures (over 350 K), resulting in a substantial increase in volume and, thus, a lower ability to form HBs; thus, the HB densities decrease. Such HB behavior with temperature in the system could corroborate the hyperbolic plot of PVT as a function of temperature as the slope of the plot gets steeper as temperature increases. Furthermore, an estimation of the change in the nonbonded potential energy during cooling down (see Figure 10) indicates that both Coulombic and van der Waals potential increase with temperature; however, the dominant factor to the change in the potential energy shifts from Coulombic to van der Waals potential as the DS increases from 0 to 3. This dominance of the Coulombic potential over van der Waals potential in the change of potential energy or vice versa is associated with the HB density of the systems.

Figure S9 in the Supporting Information shows the non-normalized HB density as a function of temperature for the models (DS = 0, 1, 2, and 3). While the HB densities of all systems (U_ds0, R_ds1, R_ds2, and U_ds3) decreased by 30–40% over a temperature range from 125 to 650 K (Figure 9b), the HB density of the U_ds0 system (DS = 0, HB density = 7.48 nm^–3^) at 650 K was still higher than that of other systems with a higher DS (e.g., 3.09, 1.13, and 0.034 nm^–3^ for DS = 1, 2, and 3, respectively); see Figure S9. This indicates that high HB density in the EC systems with low DS resulted in a less steep slope of the PVT curve (Figure 9a). From these results, we could conclude that for EC systems with a higher HB density, the locking of the system will be more pronounced, and thus an increase in the temperature would lead to smaller changes of the specific volumes, implying high *T*_g_, whereas lower HB interactions make the system more prone to change the specific volume responding to the temperature.

#### 3.2.3 Comparison between Random Models and Uniform Models

**Figure 11:**
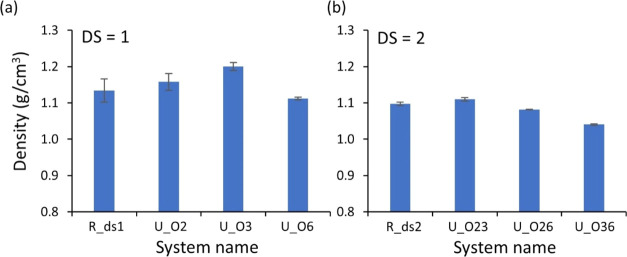
Comparison of densities of random and uniform models for DS = 1 (a) and 2 (b).

The random models were compared to the uniform models to clarify the effects of the DS and the location of the substituents on the EC systems. First, the densities of random and uniform models were compared, as shown in Figure 11. The density of the random model with DS = 1 (R_ds1, 1.13 g/cm^3^) was similar to the density of the three uniform models for DS = 1 (1.16, 1.15, and 1.20 g/cm^3^ for U _O2, U _O3, and U_O6, respectively; Figure 11a). Similarly, for models with a DS = 2, the random model with DS = 2 (R_ds2) had a similar density, 1.10 g/cm^3^, to the three uniform models with a DS = 2 (1.11, 1.11, and 1.08 g/cm^3^ for U_O23, U_O26, and U_O36, respectively); see Figure 11b. This similarity between random and uniform models for a given DS was also the case for the HB density, *R*_ee_, and *R*_g_ (Figure S10).

**Figure 12:**
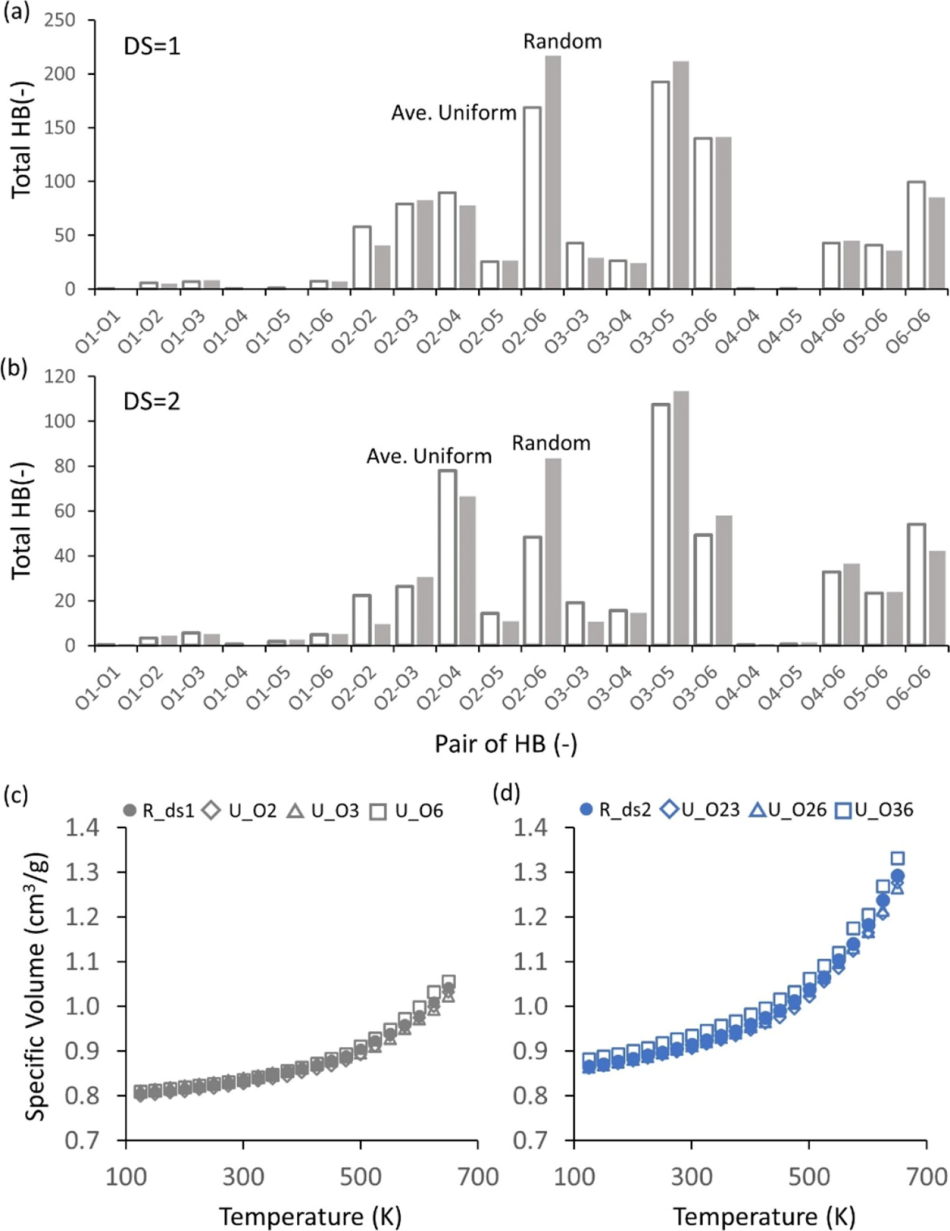
(a, b) Comparison of pairwise HB distributions between the average over the three uniform models (white) and random (gray) for (a) DS = 1 and (b) DS = 2. (c, d) Comparison of PVT curves of random and uniform models for DS = 1 (c) and 2 (d).

Since the pairwise HB distribution of uniform models varies a lot depending on the location of substituents, as elaborated in Section 3.1 (see Figure 4), it makes sense that the pairwise HB distribution of random models appears differently from any uniform models. On the other hand, it is worth noting that the pairwise HB distribution averaged over three uniform models showed a similar distribution to the random model, as shown in Figure 12a,b for both DS = 1 and 2, respectively. This similarity between uniform and random models was also observed for PVT curves, indicating that they have similar *T*_g_ values (Figure 12c,d).

Through comprehensive comparisons between random and uniform models, considering density, HBs, *R*_ee_, *R*_g_, and the PVT curve, it appears that the properties of EC with a given DS remain within a close range, irrespective of the locations of substitutions (Figures 11, 12, and S10). Therefore, it could be concluded that the distribution of substitution has a smaller effect on the properties of EC systems than that of the DS.

#### 3.2.4 Random Model Consisting of Uniformly Substituted Chains

**Figure 13:**
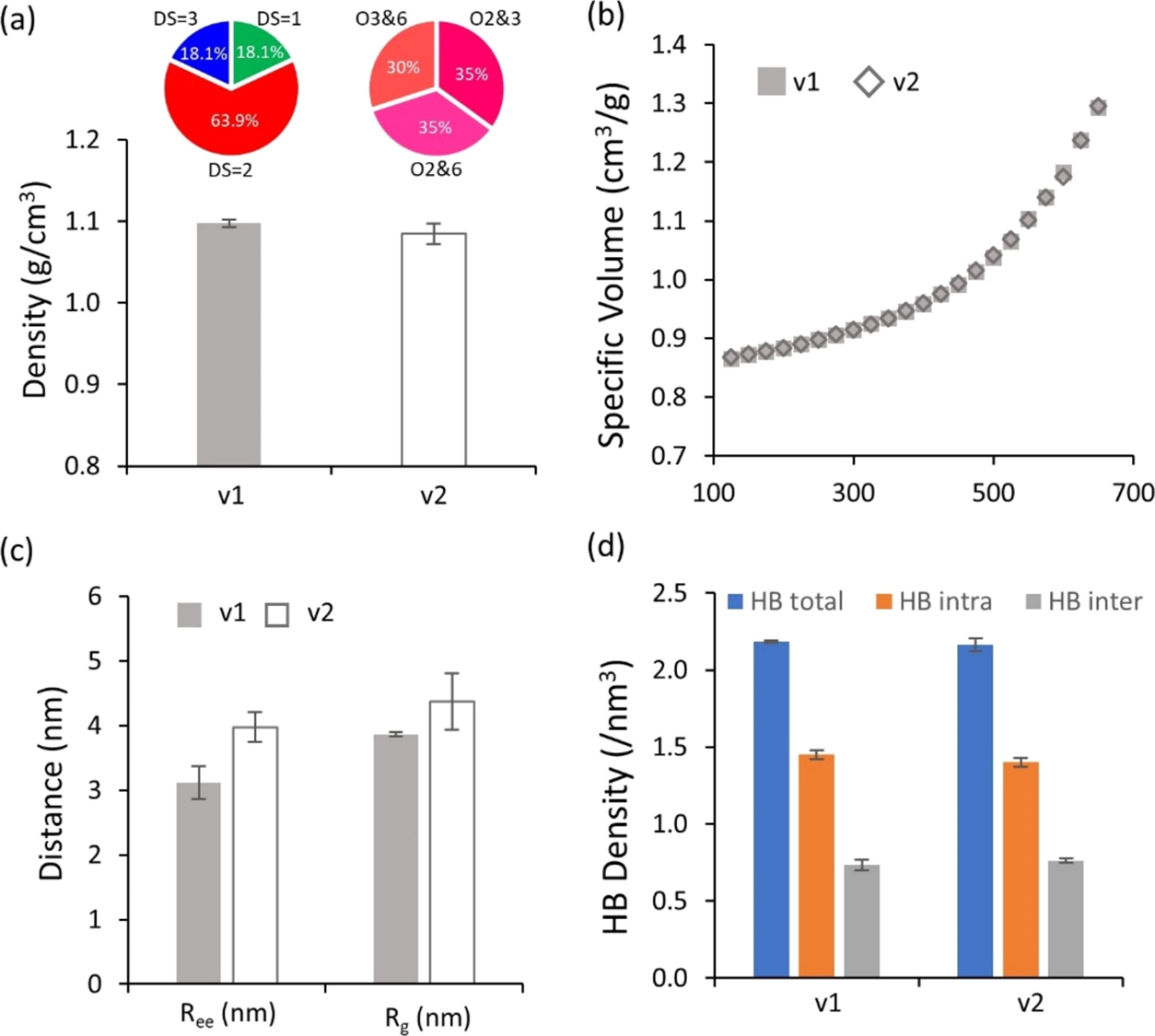
Comparison of the effect of the two random models v1 and v2 with DS = 2 in terms of (a) density, (b) PVT curves, (c) *R*_ee_ and *R*_g_, and (d) HB density. The pie charts in panel (a) exhibit the fraction of substitution types for two random models with DS = 2.

This section intends to explore the influence of the substituent locations when DS = 2 by comparing the two systems with different types of random models (see Figures 13a and S11):

- System-version 1(v1): polymer chains with a random substitution occurring both along the repeating units in chains and across the system (note: this system is identical to R_ds2),
- System-version 2(v2): a system based on a mixture of the uniformly substituted chains with DS = 2 (U_O23, U_O26, and U_O36 polymer chains).

The comparison between two random models (v1 vs v2) demonstrated, to a large extent, similar results in terms of density, the PVT curve, and HB density (see Figure 13a,b,d), while *R*_ee_ and *R*_g_ were somewhat higher for the uniformly substituted v2 than v1. These results appear very similar to the comparison between the random and uniform models described in Section 3.2.3. As the location of substituents could cause differences in inter- and intramolecular interactions of the EC system, its properties can vary depending on the location of substitution. Nonetheless, the main conclusion of this comparison is that v1 and v2 gave similar properties even if the distributions of substitution were different.

Consequently, the discussion in the current section (i.e., the comparison between two different types of random models) confirms the conclusion obtained from the comparison between uniform and random models (Section 3.2.3) that the DS has the predominant impact on the properties of EC systems, while the location of the substituents has a smaller impact on it.

### 3.3 Prediction of the Properties of EC with Respect to the DS

Based on the findings that the DS has the dominant impact on the properties of EC, we hypothesize that a property for a particular EC chain with a specified DS can be predicted (*P*_ds_^Pred.^) by making a sum of the weighted contribution of properties *x*_ds,*t*_*P*_ds,*t*_ by
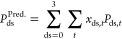
2where *P*_ds_^Pred.^ is the property of the system to be predicted and *P*_ds,*t*_ is the property of a uniform model for a DS = 0, 1, 2, and 3, which means that for a DS = 1, there are three types (U_O2, U_O3, U_O6) and for a DS = 2, there are three other types (U_O23, U_O26, U_O36); see Table S1. *x*_ds,*t*_ is the molar fraction of the corresponding type of uniform substitution in the target system. There is only one type of uniform system for DS = 0 and 3.

**Figure 14:**
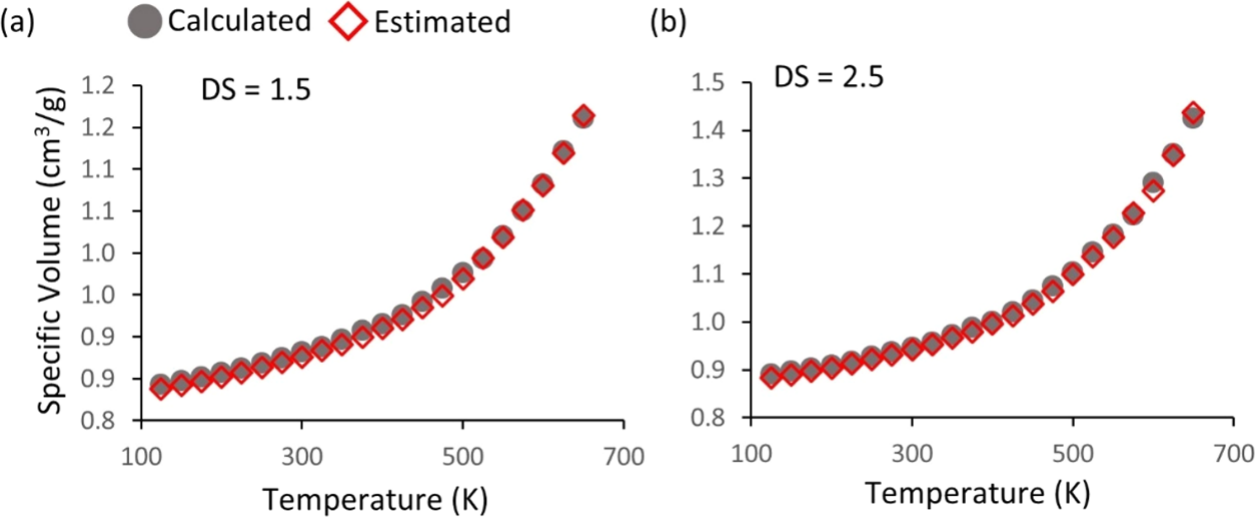
PVT curve random models with a DS = 1.5 (a) and 2.5 (b) resulting from the MD simulation and prediction by eq 2. The MD results and predicted values are indicated by gray solid circles and red empty diamonds, respectively.

The properties of random models with DS = 1.5 and 2.5 (see Figure S12 for the molar fractions of substitution types) simulated as described in Section 3.2, with predicted results based on eq 2. A good agreement was found between the specific volumes calculated by eq 2 and those obtained from the MD simulations (Figure 14). Figure S13 shows that the predicted PVT curves as per eq 2 for DS = 1 and 2 random models were identical to the simulated results. To test if this could be extended further to properties other than PVT data, other properties such as the density, total HB density, and *R*_ee_ and *R*_g_ were calculated and compared (Figure S14). In general, good agreements were obtained between the calculated and simulated data, but the agreement was the least good for the total HB density for a DS = 2.5. This can be explained by the fact that, at a DS = 2.5, only a few unsubstituted hydroxyl groups are available to create HBs, which makes the predictions and simulations more sensitive to fluctuation.

Building on these significant findings, we concluded that for EC systems with any DS, the *T*_g_ or any other properties in Figure S13 could be reliably predicted using data from uniform models. From the fact that the total number of hydrogen bonds (HBs) is largely maintained, Figure 14b, regardless of the substitution type as long as the degree of substitution (DS) is the same, as shown in Figure S15; we infer that the linear function predicts the properties well, especially density properties such as the PVT data curve. However, it provides a somewhat rough prediction of *R*_ee_ and *R*_g_, which are more affected by accumulated local changes, such as dihedral angles, than the former properties.

It would have been interesting to validate the approach presented in eq 2 against experimental data, but we could not systematically access sequentially substituted ethylcellulose. However, if one speculates, the model can work only if the uniform and random chains do not interact differently. Another challenge will arise if one has, for example, hydroxypropyl groups, which are reactive themselves and can form chains of substituents. This will cause difficulties in knowing the *x*_ds,*t*_ and covering all possible *P*_ds,*t*_.

This groundbreaking discovery introduces a new, efficient approach to predicting material properties for cellulose derivatives quickly and easily. The approach requires a given DS and available simulated data for the uniform models (requiring simulations only once for each specific cellulose derivative); if these are available, then the prediction process can be executed with high precision. This advancement opens vast possibilities, such as enabling the pharmaceutical industry to predict properties swiftly for incoming EC batches. In addition, it allows predictions of properties of EC and potentially other cellulose derivatives via a simple combination of a linear function and MD simulations. In the future, it might be possible that the generated properties can be utilized as input data in material libraries, where machine learning algorithms can categorize and use them for material design and for new applications.

## 4 Conclusions

By using MD simulations, the effects of the DS and location of substituents on the properties of EC such as density, HB formation, *R*_ee_, *R*_g_, and the PVT curve were explored. To get a detailed understanding of the variation in the DS and locations of the substituents in the repeating unit, uniformly substituted EC systems with varying DS (= 0, 1, 2, 3) were first studied. Uniformly substituted models refer to EC chains exclusively substituted at specific positions among carbons 2, 3, and 6, (i.e., a given DS for all glucose units in the system). Subsequently, the random models of EC systems with varying DS (0, 1, 2, 3), where the repeating glucose units of the chains to which a specific DS is randomly assigned, were simulated and discussed compared to the uniform models.

The detailed analysis of the pairwise HB distribution in the uniform models elucidates that the presence or absence of substitutions on the O3 and O6 positions have a crucial effect on forming intramolecular and intermolecular HBs, respectively. Our simulation results concerning density, HB density, *R*_ee_, and *R*_g_ show that, while these properties change depending on the location of substituents, the DS of the system pursues a dominant influence on them. Thus, the properties of the EC system are primarily determined by its DS, whether random substitution or other methods were used to achieve the target DS.

For both uniform and random models, as the DS of the EC system increases, its density and HB density decrease, while *R*_ee_ and *R*_g_ increase. These results indicate that an increase in the DS of the EC system weakens the inter- and intramolecular interactions (decreases HB density) and the ethyl modification increases the chain stericity, thereby making the chains straighter (i.e., increase in *R*_ee_ and *R*_g_), consequently leading to a less densely packed EC system. Furthermore, a qualitative comparison of PVT curves for different DS demonstrates that the increase in specific volume leads to a decrease in the *T*_g_ of the EC system with increasing DS, which is attributed to the decrease in inter- and intramolecular interactions and increased possibility for the chains to move with increasing DS.

Finally, based on our findings that the dominant effect on the EC system is the DS, we propose that it is possible to predict the properties of an amorphous EC system with a given DS by a simple linear function, which sums the property of the uniform model multiplied by its molar fraction for the given EC system. We corroborated this idea by comparing the simulation results of the EC system with DS = 2.5 and 1.5 with predicted results using the properties of uniform models (U_ds0, U_O2, U_O3, U_O6, U_O23, U_O26, U_36, and U_ds3) in terms of density, HB density, *R*_ee_, and *R*_g_.

We hope that the outcomes of our simulation study will offer fresh insights into exploring the properties of cellulose derivatives and spur the field to utilize cellulose derivatives instead of fossil-based materials.

## Supporting Information Available

Additional simulation details, a description of possible donor and acceptor pairs, complementary results regarding Tg, PVT curves, atom distances, HB, HB densities, distribution of the dihedral angles, end-to-end distances, and radius of gyrations (PDF)
