# Supplementary material for: In-Depth Understanding of the Effect of the Distribution of Substituents on the Morphology and Physical Properties of Ethylcellulose: Molecular Dynamics Simulations Insights
Source: Biomacromolecules. 2024 Jun 24;25(7):4046–62. doi: 10.1021/acs.biomac.4c00166 (PMC11238332; doi:10.1021/acs.biomac.4c00166)
Supplement: Supplementary file 1 — bm4c00166_si_001.pdf [file bm4c00166_si_001.pdf]

In-depth understanding of the effect of the distribution of substituents on the morphology and physical properties of ethylcellulose: Molecular dynamics simulations insights

Donghyun Kim[1,2,6], Patric Elf[3,4], Fritjof Nilsson[3,4,7], Mikael S. Hedenqvist[3,4,5], Anette Larsson[1,2,6]*

[1] Applied Chemistry, Department of Chemistry and Chemical Engineering, Chalmers University of Technology, SE-412 96 Gothenburg, Sweden

[2] FibRe Centre for Lignocellulose-based Thermoplastics, Department of Chemistry and Chemical Engineering, Chalmers University of Technology, SE-412 96 Gothenburg, Sweden

[3] Department of Fibre and Polymer Technology, School of Engineering Sciences in Chemistry, Biotechnology and Health, KTH Royal Institute of Technology, SE-100 44 Stockholm, Sweden

[4] FibRe Vinnova competence center, KTH Royal Institute of Technology, Stockholm, Sweden

[5] Wallenberg Wood Science Center, KTH Royal Institute of Technology, Stockholm, Sweden

[6] Wallenberg Wood Science Center, Chalmers University of Technology, Gothenburg, Sweden

[7] FSCN research centre, Mid Sweden University, 85170 Sundsvall, Sweden

20 Table S1. Description of ethylcellulose systems on the DS and the location of substituents.

System Name	Degree of Substitution	Substitution Type	Position of Substitution
U_ds0	0	Uniform	-
U_O2	1	Uniform	All O2
U_O3	1	Uniform	All O3
U_O6	1	Uniform	All O6
U_O23	2	Uniform	All O2 & O3
U_O26	2	Uniform	All O2 & O6
U_O36	2	Uniform	All O3 & O6
U_ds3	3	Uniform	All O2 & O3 & O6
R_ds1	1	Random	O2 or O3 or O6
R_ds1.5	1.5	Random	O2 or O3 or O6
R_ds2 (R_ds2(v1))	2	Random	O2 or O3 or O6
R_ds2(v2)	2	Random	O2 or O3 or O6 (uniform chains)
R_ds2.5	2.5	Random	O2 or O3 or O6

21

Table S2. The total number of anhydroglucose units with DS = 0, 1, 2, and 3 in investigated EC systems. The sum of a system should be 720.

**System Name**	**Degree of Substitution**			
	0	1	2	3
U_ds0	720	0	0	0
U_O2	0	720	0	0
U_O3	0	720	0	0
U_O6	0	720	0	0
U_O23	0	0	720	0
U_O26	0	0	720	0
U_O36	0	0	720	0
U_ds3	0	0	0	720
R_ds1	20	690	0	10
R_ds1.5	0	360	360	0
R_ds2(v1)	0	130	460	130
R_ds2(v2)	0	0	720	0
R_ds2.5	0	90	180	450

Table S3. Description of 21-step decompression to result in amorphous EC systems.

Step	Ensemble	Pressure (bar)	Temperature (K)	Duration (ns)
1	NVT		600	0.50
2	NVT		300	0.25
3	NPT	0.2 Pmax	300	0.50
4,5	NVT		600, 300	0.25, 0.50
6	NPT	0.6 Pmax	300	0.25
7,8	NVT		600, 300	0.50, 0.50
9	NPT	Pmax	300	0.50, *(2.0)
10,11	NVT		600, 300	0.25, 0.50
12	NPT	0.5 Pmax	300	0.050
13,14	NVT		600, 300	0.025, 0.050
15	NPT	0.1 Pmax	300	0.025
16,17	NVT		600, 300	0.025, 0.050
18	NPT	0.01 Pmax	300	0.025
19,20	NVT		600, 300	0.025, 0.050
21	NPT	1	300	20

- The duration of step 9 is 2 ns for pure cellulose system (DS = 0)
- Pmax = 800 bar for pure cellulose systems. Pmax is 200 bar for other systems.

We exploited modified CHARMM36-jul2021 force field to simulate pure cellulose and ethylcellulose systems. However, there are some parameters which CHARMM36-jul2021 does not recognize. Thus, it needs to add such information to the “carb.rtp” and “ffbonded.itp” files of it. To this end, we prepared eight uniformly substituted chains consisting of three repeating units that are referred to as O1T (edge unit ending with O1 at carbon 1), MGC (middle unit), and O4T (edge unit ending with O4 at carbon 4) in the present work (i.e., 8 types of chains with DS = 0, 1 (O2, O3, O6), 2(O23, O26, O36), 3). Via CGenFF server, we can obtain all new topology information for above chains including atomtypes, charges, bond connectivity, and potential parameters. Table S4 exhibits some information of 8 chains obtained from CGenFF server. All force field parameters of randomly substituted ethylcellulose chains can be assigned by the information of O1T, MGC and O4T units of corresponding substitution type.

Table S4. Atomtype and atomic charge obtained from CGenFF server for uniformly substitute EC chains with DS equal to 0, 1, 2, and 3 consisting of three repeating units that are O1T (edge unit ending with O1 at carbon 1), MGC (middle unit), and O4T (edge unit ending with O4 at carbon 4).

DS = 0								
O4T			MGC			O1T		
Atom	Atomtype	Charge	Atom	Atomtype	Charge	Atom	Atomtype	Charge
C5	CG311	0.062	C5	CG311	0.053	C5	CG311	0.053
H5	HGA1	0.09	H5	HGA1	0.09	H5	HGA1	0.09
O5	OG3C61	-0.4	O5	OG3C61	-0.4	O5	OG3C61	-0.401
C1	CG311	0.272	C1	CG311	0.272	C1	CG311	0.308
H1	HGA1	0.09	H1	HGA1	0.09	H1	HGA1	0.09
O2	OG311	-0.646	O2	OG311	-0.646	O1	OG311	-0.65
HO2	HGP1	0.419	HO2	HGP1	0.419	HO1	HGP1	0.416
C2	CG311	0.16	C2	CG311	0.165	O2	OG311	-0.645
H2	HGA1	0.09	H2	HGA1	0.09	HO2	HGP1	0.419
C3	CG311	0.149	C3	CG311	0.145	C2	CG311	0.169
H3	HGA1	0.09	H3	HGA1	0.09	H2	HGA1	0.09
HO3	HGP1	0.419	HO3	HGP1	0.419	C3	CG311	0.14
O3	OG311	-0.642	O3	OG311	-0.643	HO3	HGP1	0.419
C4	CG311	0.147	C4	CG311	0.124	O3	OG311	-0.643
H4	HGA1	0.09	H4	HGA1	0.09	H3	HGA1	0.09
O4	OG311	-0.645	O4	OG301	-0.402	C4	CG311	0.124
HO4	HGP1	0.419	C6	CG321	0.091	H4	HGA1	0.09
C6	CG321	0.082	H61	HGA2	0.09	O4	OG301	-0.402
H61	HGA2	0.09	O6	OG311	-0.646	C6	CG321	0.091
O6	OG311	-0.646	H62	HGA2	0.09	H61	HGA2	0.09
H62	HGA2	0.09	HO6	HGP1	0.419	O6	OG311	-0.646
HO6	HGP1	0.419				H62	HGA2	0.09
						HO6	HGP1	0.419
DS = 1 (O2)								
O4T			MGC			O1T		
Atom	Atomtype	Charge	Atom	Atomtype	Charge	Atom	Atomtype	Charge
C5	CG311	0.062	C5	CG311	0.053	C5	CG311	0.053
H5	HGA1	0.09	H5	HGA1	0.09	H5	HGA1	0.09

O5	OG3C61	-0.4	O5	OG3C61	-0.4	O5	OG3C61	-0.401
C1	CG311	0.266	C1	CG311	0.266	C1	CG311	0.303
H1	HGA1	0.09	H1	HGA1	0.09	H1	HGA1	0.09
C2	CG311	0.142	C2	CG311	0.147	O1	OG311	-0.651
H2	HGA1	0.09	H2	HGA1	0.09	HO1	HGP1	0.416
O2	OG301	-0.373	O2	OG301	-0.373	C2	CG311	0.152
C3	CG311	0.145	C3	CG311	0.141	H2	HGA1	0.09
H3	HGA1	0.09	H3	HGA1	0.09	O2	OG301	-0.373
O3	OG311	-0.643	O3	OG311	-0.644	C3	CG311	0.136
HO3	HGP1	0.419	HO3	HGP1	0.419	H3	HGA1	0.09
HO4	HGP1	0.419	O4	OG301	-0.402	O3	OG311	-0.644
O4	OG311	-0.645	C4	CG311	0.129	HO3	HGP1	0.419
H4	HGA1	0.09	H4	HGA1	0.09	O4	OG301	-0.402
C4	CG311	0.152	C6	CG321	0.091	H4	HGA1	0.09
C6	CG321	0.082	H61	HGA2	0.09	C4	CG311	0.129
H61	HGA2	0.09	O6	OG311	-0.646	C6	CG321	0.091
O6	OG311	-0.646	H62	HGA2	0.09	H61	HGA2	0.09
H62	HGA2	0.09	HO6	HGP1	0.419	O6	OG311	-0.646
HO6	HGP1	0.419	HA2	HGA3	0.09	H62	HGA2	0.09
C9	CG321	-0.013	C9	CG321	-0.013	HO6	HGP1	0.419
H91	HGA2	0.09	H91	HGA2	0.09	C9	CG321	-0.013
H92	HGA2	0.09	H92	HGA2	0.09	H91	HGA2	0.09
C10	CG331	-0.267	C10	CG331	-0.267	H92	HGA2	0.09
HA1	HGA3	0.09	HA1	HGA3	0.09	C10	CG331	-0.267
HA2	HGA3	0.09	HA3	HGA3	0.09	HA1	HGA3	0.09
HA3	HGA3	0.09				HA2	HGA3	0.09
						HA3	HGA3	0.09
DS = 1 (O3)								
O4T			MGC			O1T		
Atom	Atomtype	Charge	Atom	Atomtype	Charge	Atom	Atomtype	Charge
C5	CG311	0.067	C5	CG311	0.058	C5	CG311	0.058
H5	HGA1	0.09	H5	HGA1	0.09	H5	HGA1	0.09
O5	OG3C61	-0.4	O5	OG3C61	-0.4	O5	OG3C61	-0.401
C1	CG311	0.277	C1	CG311	0.277	C1	CG311	0.313
H1	HGA1	0.09	H1	HGA1	0.09	H1	HGA1	0.09
C2	CG311	0.156	C2	CG311	0.161	O1	OG311	-0.65
H2	HGA1	0.09	H2	HGA1	0.09	HO1	HGP1	0.416
O2	OG311	-0.647	O2	OG311	-0.647	C2	CG311	0.165
HO2	HGP1	0.419	HO2	HGP1	0.419	H2	HGA1	0.09
C3	CG311	0.129	C3	CG311	0.124	O2	OG311	-0.646
H3	HGA1	0.09	H3	HGA1	0.09	HO2	HGP1	0.419
O3	OG301	-0.373	O3	OG301	-0.373	C3	CG311	0.119

HO4	HGP1	0.419	O4	OG301	-0.402	H3	HGA1	0.09
O4	OG311	-0.646	C4	CG311	0.119	O3	OG301	-0.373
H4	HGA1	0.09	H4	HGA1	0.09	O4	OG301	-0.402
C4	CG311	0.143	C6	CG321	0.091	H4	HGA1	0.09
C6	CG321	0.082	H61	HGA2	0.09	C4	CG311	0.119
H61	HGA2	0.09	O6	OG311	-0.646	C6	CG321	0.091
O6	OG311	-0.646	H62	HGA2	0.09	H61	HGA2	0.09
H62	HGA2	0.09	HO6	HGP1	0.419	O6	OG311	-0.646
HO6	HGP1	0.419	HC2	HGA3	0.09	H62	HGA2	0.09
C11	CG321	-0.013	C11	CG321	-0.013	HO6	HGP1	0.419
HB1	HGA2	0.09	HB1	HGA2	0.09	C11	CG321	-0.013
HB2	HGA2	0.09	HB2	HGA2	0.09	HB1	HGA2	0.09
C12	CG331	-0.267	C12	CG331	-0.267	HB2	HGA2	0.09
HC1	HGA3	0.09	HC1	HGA3	0.09	C12	CG331	-0.267
HC2	HGA3	0.09	HC3	HGA3	0.09	HC1	HGA3	0.09
HC3	HGA3	0.09				HC2	HGA3	0.09
						HC3	HGA3	0.09
DS = 1 (O6)								
O4T			MGC			O1T		
Atom	Atomtype	Charge	Atom	Atomtype	Charge	Atom	Atomtype	Charge
C5	CG311	0.046	C5	CG311	0.037	C5	CG311	0.037
H5	HGA1	0.09	H5	HGA1	0.09	H5	HGA1	0.09
O5	OG3C61	-0.4	O5	OG3C61	-0.4	O5	OG3C61	-0.401
C1	CG311	0.272	C1	CG311	0.272	C1	CG311	0.308
H1	HGA1	0.09	H1	HGA1	0.09	H1	HGA1	0.09
C2	CG311	0.16	C2	CG311	0.165	O1	OG311	-0.65
H2	HGA1	0.09	H2	HGA1	0.09	HO1	HGP1	0.416
O2	OG311	-0.646	O2	OG311	-0.646	C2	CG311	0.169
HO2	HGP1	0.419	HO2	HGP1	0.419	H2	HGA1	0.09
C3	CG311	0.149	C3	CG311	0.145	O2	OG311	-0.645
H3	HGA1	0.09	H3	HGA1	0.09	HO2	HGP1	0.419
O3	OG311	-0.642	O3	OG311	-0.643	C3	CG311	0.14
HO3	HGP1	0.419	HO3	HGP1	0.419	H3	HGA1	0.09
HO4	HGP1	0.419	O4	OG301	-0.402	O3	OG311	-0.643
O4	OG311	-0.645	H4	HGA1	0.09	HO3	HGP1	0.419
H4	HGA1	0.09	C4	CG311	0.138	H4	HGA1	0.09
C4	CG311	0.161	C6	CG321	0.059	C4	CG311	0.138
C6	CG321	0.05	H61	HGA2	0.09	O4	OG301	-0.402
H61	HGA2	0.09	O6	OG301	-0.363	C6	CG321	0.059
O6	OG301	-0.363	H62	HGA2	0.09	H61	HGA2	0.09
H62	HGA2	0.09	C7	CG321	-0.013	O6	OG301	-0.363
C7	CG321	-0.013	H71	HGA2	0.09	H62	HGA2	0.09

H71	HGA2	0.09	H72	HGA2	0.09	H82	HGA3	0.09
H72	HGA2	0.09	C8	CG331	-0.267	C7	CG321	-0.013
C8	CG331	-0.267	H81	HGA3	0.09	H71	HGA2	0.09
H81	HGA3	0.09	H82	HGA3	0.09	H72	HGA2	0.09
H82	HGA3	0.09	H83	HGA3	0.09	C8	CG331	-0.267
H83	HGA3	0.09				H81	HGA3	0.09
						H83	HGA3	0.09
DS = 2 (O23)								
O4T			MGC			O1T		
Atom	Atomtype	Charge	Atom	Atomtype	Charge	Atom	Atomtype	Charge
C5	CG311	0.067	C5	CG311	0.058	C5	CG311	0.058
H5	HGA1	0.09	H5	HGA1	0.09	H5	HGA1	0.09
O5	OG3C61	-0.4	O5	OG3C61	-0.4	O5	OG3C61	-0.401
C1	CG311	0.271	C1	CG311	0.271	C1	CG311	0.308
H1	HGA1	0.09	H1	HGA1	0.09	H1	HGA1	0.09
C2	CG311	0.137	C2	CG311	0.142	O1	OG311	-0.651
H2	HGA1	0.09	H2	HGA1	0.09	HO1	HGP1	0.416
O2	OG301	-0.373	O2	OG301	-0.373	O2	OG301	-0.373
O3	OG301	-0.373	C3	CG311	0.119	H2	HGA1	0.09
C3	CG311	0.124	H3	HGA1	0.09	C2	CG311	0.147
H3	HGA1	0.09	O3	OG301	-0.373	C3	CG311	0.114
C4	CG311	0.148	C4	CG311	0.124	H3	HGA1	0.09
H4	HGA1	0.09	H4	HGA1	0.09	O3	OG301	-0.373
O4	OG311	-0.646	O4	OG301	-0.402	C4	CG311	0.124
HO4	HGP1	0.419	HO6	HGP1	0.419	H4	HGA1	0.09
HO6	HGP1	0.419	H62	HGA2	0.09	O4	OG301	-0.402
H62	HGA2	0.09	O6	OG311	-0.646	HO6	HGP1	0.419
H61	HGA2	0.09	H61	HGA2	0.09	H62	HGA2	0.09
C6	CG321	0.082	C6	CG321	0.091	H61	HGA2	0.09
O6	OG311	-0.646	C9	CG321	-0.013	C6	CG321	0.091
C9	CG321	-0.013	H91	HGA2	0.09	O6	OG311	-0.646
H91	HGA2	0.09	H92	HGA2	0.09	C9	CG321	-0.013
H92	HGA2	0.09	C10	CG331	-0.267	H91	HGA2	0.09
C10	CG331	-0.267	HA1	HGA3	0.09	H92	HGA2	0.09
HA1	HGA3	0.09	HA2	HGA3	0.09	C10	CG331	-0.267
HA2	HGA3	0.09	HA3	HGA3	0.09	HA1	HGA3	0.09
HA3	HGA3	0.09	C11	CG321	-0.013	HA2	HGA3	0.09
HC2	HGA3	0.09	HB1	HGA2	0.09	HA3	HGA3	0.09
C11	CG321	-0.013	HB2	HGA2	0.09	HC2	HGA3	0.09
HB1	HGA2	0.09	C12	CG331	-0.267	C11	CG321	-0.013
HB2	HGA2	0.09	HC1	HGA3	0.09	HB1	HGA2	0.09
C12	CG331	-0.267	HC2	HGA3	0.09	HB2	HGA2	0.09

HC1	HGA3	0.09	HC3	HGA3	0.09	C12	CG331	-0.267
HC3	HGA3	0.09				HC1	HGA3	0.09
						HC3	HGA3	0.09
DS = 2 (O26)								
O4T			MGC			O1T		
Atom	Atomtype	Charge	Atom	Atomtype	Charge	Atom	Atomtype	Charge
C5	CG311	0.046	C5	CG311	0.037	C5	CG311	0.037
H5	HGA1	0.09	H5	HGA1	0.09	H5	HGA1	0.09
O5	OG3C61	-0.4	O5	OG3C61	-0.4	O5	OG3C61	-0.401
C1	CG311	0.266	C1	CG311	0.266	C1	CG311	0.303
H1	HGA1	0.09	H1	HGA1	0.09	H1	HGA1	0.09
C2	CG311	0.142	C2	CG311	0.147	O1	OG311	-0.651
H2	HGA1	0.09	H2	HGA1	0.09	HO1	HGP1	0.416
O2	OG301	-0.373	O2	OG301	-0.373	O2	OG301	-0.373
O3	OG311	-0.643	C3	CG311	0.141	H2	HGA1	0.09
C3	CG311	0.145	H3	HGA1	0.09	C2	CG311	0.152
HO3	HGP1	0.419	O3	OG311	-0.644	C3	CG311	0.136
H3	HGA1	0.09	HO3	HGP1	0.419	H3	HGA1	0.09
C4	CG311	0.166	C4	CG311	0.143	O3	OG311	-0.644
H4	HGA1	0.09	H4	HGA1	0.09	HO3	HGP1	0.419
O4	OG311	-0.645	O4	OG301	-0.402	C4	CG311	0.143
HO4	HGP1	0.419	H62	HGA2	0.09	H4	HGA1	0.09
H62	HGA2	0.09	O6	OG301	-0.363	O4	OG301	-0.402
H61	HGA2	0.09	H61	HGA2	0.09	H62	HGA2	0.09
C6	CG321	0.05	C6	CG321	0.059	H61	HGA2	0.09
O6	OG301	-0.363	C7	CG321	-0.013	C6	CG321	0.059
C7	CG321	-0.013	H71	HGA2	0.09	O6	OG301	-0.363
H71	HGA2	0.09	H72	HGA2	0.09	C7	CG321	-0.013
H72	HGA2	0.09	C8	CG331	-0.267	H71	HGA2	0.09
C8	CG331	-0.267	H81	HGA3	0.09	H72	HGA2	0.09
H81	HGA3	0.09	H82	HGA3	0.09	C8	CG331	-0.267
H82	HGA3	0.09	H83	HGA3	0.09	H81	HGA3	0.09
H83	HGA3	0.09	C9	CG321	-0.013	H82	HGA3	0.09
HA2	HGA3	0.09	H91	HGA2	0.09	H83	HGA3	0.09
C9	CG321	-0.013	H92	HGA2	0.09	HA2	HGA3	0.09
H91	HGA2	0.09	C10	CG331	-0.267	C9	CG321	-0.013
H92	HGA2	0.09	HA1	HGA3	0.09	H91	HGA2	0.09
C10	CG331	-0.267	HA2	HGA3	0.09	H92	HGA2	0.09
HA1	HGA3	0.09	HA3	HGA3	0.09	C10	CG331	-0.267
HA3	HGA3	0.09				HA1	HGA3	0.09
						HA3	HGA3	0.09
DS = 2 (O36)								

O4T			MGC			O1T		
Atom	Atomtype	Charge	Atom	Atomtype	Charge	Atom	Atomtype	Charge
C5	CG311	0.051	C5	CG311	0.042	C5	CG311	0.042
H5	HGA1	0.09	H5	HGA1	0.09	H5	HGA1	0.09
O5	OG3C61	-0.4	O5	OG3C61	-0.4	O5	OG3C61	-0.401
C1	CG311	0.277	C1	CG311	0.277	C1	CG311	0.313
H1	HGA1	0.09	H1	HGA1	0.09	H1	HGA1	0.09
C2	CG311	0.156	C2	CG311	0.161	O1	OG311	-0.65
H2	HGA1	0.09	H2	HGA1	0.09	HO1	HGP1	0.416
O2	OG311	-0.647	O2	OG311	-0.647	O2	OG311	-0.646
HO2	HGP1	0.419	HO2	HGP1	0.419	H2	HGA1	0.09
O3	OG301	-0.373	C3	CG311	0.124	C2	CG311	0.165
C3	CG311	0.129	H3	HGA1	0.09	HO2	HGP1	0.419
H3	HGA1	0.09	O3	OG301	-0.373	C3	CG311	0.119
C4	CG311	0.157	C4	CG311	0.133	H3	HGA1	0.09
H4	HGA1	0.09	H4	HGA1	0.09	O3	OG301	-0.373
O4	OG311	-0.646	O4	OG301	-0.402	C4	CG311	0.133
HO4	HGP1	0.419	H62	HGA2	0.09	H4	HGA1	0.09
H62	HGA2	0.09	O6	OG301	-0.363	O4	OG301	-0.402
H61	HGA2	0.09	H61	HGA2	0.09	H62	HGA2	0.09
C6	CG321	0.05	C6	CG321	0.059	H61	HGA2	0.09
O6	OG301	-0.363	C7	CG321	-0.013	C6	CG321	0.059
C7	CG321	-0.013	H71	HGA2	0.09	O6	OG301	-0.363
H71	HGA2	0.09	H72	HGA2	0.09	C7	CG321	-0.013
H72	HGA2	0.09	C8	CG331	-0.267	H71	HGA2	0.09
C8	CG331	-0.267	H81	HGA3	0.09	H72	HGA2	0.09
H81	HGA3	0.09	H82	HGA3	0.09	C8	CG331	-0.267
H82	HGA3	0.09	H83	HGA3	0.09	H81	HGA3	0.09
H83	HGA3	0.09	C11	CG321	-0.013	H82	HGA3	0.09
HC2	HGA3	0.09	HB1	HGA2	0.09	H83	HGA3	0.09
C11	CG321	-0.013	HB2	HGA2	0.09	HC2	HGA3	0.09
HB1	HGA2	0.09	C12	CG331	-0.267	C11	CG321	-0.013
HB2	HGA2	0.09	HC1	HGA3	0.09	HB1	HGA2	0.09
C12	CG331	-0.267	HC2	HGA3	0.09	HB2	HGA2	0.09
HC1	HGA3	0.09	HC3	HGA3	0.09	C12	CG331	-0.267
HC3	HGA3	0.09				HC1	HGA3	0.09
						HC3	HGA3	0.09
DS = 3								
O4T			MGC			O1T		
Atom	Atomtype	Charge	Atom	Atomtype	Charge	Atom	Atomtype	Charge
C5	CG311	0.051	C5	CG311	0.042	C5	CG311	0.042
H5	HGA1	0.09	H5	HGA1	0.09	H5	HGA1	0.09

O5	OG3C61	-0.4	O5	OG3C61	-0.4	O5	OG3C61	-0.401
C1	CG311	0.271	C1	CG311	0.271	C1	CG311	0.308
H1	HGA1	0.09	H1	HGA1	0.09	H1	HGA1	0.09
C2	CG311	0.137	C2	CG311	0.142	O1	OG311	-0.651
H2	HGA1	0.09	H2	HGA1	0.09	HO1	HGP1	0.416
O2	OG301	-0.373	O2	OG301	-0.373	C2	CG311	0.147
H3	HGA1	0.09	H3	HGA1	0.09	H2	HGA1	0.09
O3	OG301	-0.373	O3	OG301	-0.373	O2	OG301	-0.373
C3	CG311	0.124	C3	CG311	0.119	O3	OG301	-0.373
C4	CG311	0.162	C4	CG311	0.138	H3	HGA1	0.09
H4	HGA1	0.09	H4	HGA1	0.09	C3	CG311	0.114
O4	OG311	-0.646	O4	OG301	-0.402	C4	CG311	0.138
HO4	HGP1	0.419	C6	CG321	0.059	H4	HGA1	0.09
C6	CG321	0.05	H61	HGA2	0.09	O4	OG301	-0.402
H61	HGA2	0.09	O6	OG301	-0.363	C6	CG321	0.059
O6	OG301	-0.363	H62	HGA2	0.09	H61	HGA2	0.09
H62	HGA2	0.09	H82	HGA3	0.09	O6	OG301	-0.363
H82	HGA3	0.09	H83	HGA3	0.09	H62	HGA2	0.09
H83	HGA3	0.09	H81	HGA3	0.09	H83	HGA3	0.09
H81	HGA3	0.09	H71	HGA2	0.09	H82	HGA3	0.09
C7	CG321	-0.013	H72	HGA2	0.09	H81	HGA3	0.09
H72	HGA2	0.09	C7	CG321	-0.013	C7	CG321	-0.013
H71	HGA2	0.09	C8	CG331	-0.267	H72	HGA2	0.09
C8	CG331	-0.267	HA3	HGA3	0.09	H71	HGA2	0.09
HA3	HGA3	0.09	HA1	HGA3	0.09	C8	CG331	-0.267
HA1	HGA3	0.09	C10	CG331	-0.267	HA3	HGA3	0.09
C10	CG331	-0.267	HA2	HGA3	0.09	HA1	HGA3	0.09
HA2	HGA3	0.09	H91	HGA2	0.09	C10	CG331	-0.267
H91	HGA2	0.09	C9	CG321	-0.013	HA2	HGA3	0.09
C9	CG321	-0.013	H92	HGA2	0.09	H91	HGA2	0.09
H92	HGA2	0.09	HC2	HGA3	0.09	C9	CG321	-0.013
HC2	HGA3	0.09	C11	CG321	-0.013	H92	HGA2	0.09
C11	CG321	-0.013	HB1	HGA2	0.09	HC2	HGA3	0.09
HB1	HGA2	0.09	HB2	HGA2	0.09	C11	CG321	-0.013
HB2	HGA2	0.09	C12	CG331	-0.267	HB1	HGA2	0.09
C12	CG331	-0.267	HC1	HGA3	0.09	HB2	HGA2	0.09
HC1	HGA3	0.09	HC3	HGA3	0.09	C12	CG331	-0.267
HC3	HGA3	0.09				HC1	HGA3	0.09
						HC3	HGA3	0.09

When hydrogen atoms on oxygens O2, O3, and O6 are substituted with ethyl groups, the oxygens transfer from being both acceptors and donors to solely functioning as acceptors. Consequently, for unsubstituted cellulose chains, HB formation involving O2, O3, and O6 entails both acceptors and donors. However, as the degree of ethyl substitution (DS) increases, the number of potential donors decreases, thereby reducing the number of feasible HBs. This reduction is corroborated by the decline in HB count and HB density with increasing DS (see Fig. 2C). Additionally, the attachment of bulky ethyl groups poses steric hindrance, further impeding the ability of the modified oxygen to act as an acceptor. The substation of the hydrogen at one oxygen can also introduce alter positions of the other atoms, resulting in new conformations. A summary of the different oxygen pairs´ ability to act as donors and acceptors, and the effect on this ability by substitution with ethyl groups are summarized in Table S5.

Table S5 Donor and Acceptor Oxygens to form hydrogen bonds in the ethylcellulose systems. “O” and “X” denote the oxygen positions that are capable and not capable of being Donor and Acceptor, respectively. Cells highlighted in green color means that oxygen cannot contribute to forming HB as donor via substitution at corresponding position, but it can still be Acceptor.

	U_ds0		U_O2		U_O3		U_O6	
	Donor	Acceptor	D	A	D	A	D	A
O1 (end)	O	O	O	O	O	O	O	O
O2	O	O	X	O	O	O	O	O
O3	O	O	O	O	X	O	O	O
O6	O	O	O	O	O	O	X	O
391O5	X	O	X	O	X	O	X	O
O4(linkage)	X	O	X	O	X	O	X	O
O4 (end)	O	O	O	O	O	O	O	O

Even though the uncertainties with determining the T_g we can determine an onset Tg from the deviation of a linear fit using the lower temperature points. Using the 6 lowest temperature points (125-250K), we do indeed see that ethyl substitutions to the hydroxyl groups lower the T_g. The 1% deviation from this linear fit occurs between the 425K and 450K points, whereas the 1% deviation for all other ethyl cellulose systems occurs significantly lower, as can be seen in Table S6.

Table S6. The low onset is the last point before the low linear fit deviates more than 1% and high onset is the first point where the low linear fit deviates more than 1% from the temperature measured. The Intersection is the intersection between the high and low linear fits.

System	Low Onset	High Onset
U_ds0	425	450
U_O2	375	400
U_O3	375	400
U_O6	350	375
U_O23	325	350
U_O26	325	350
U_O36	325	350
U_ds3	300	325
R_ds1	350	375
R_ds1.5	350	375
R_ds2_v1	325	350
R_ds2_v2	325	350
R_ds2.5	325	350

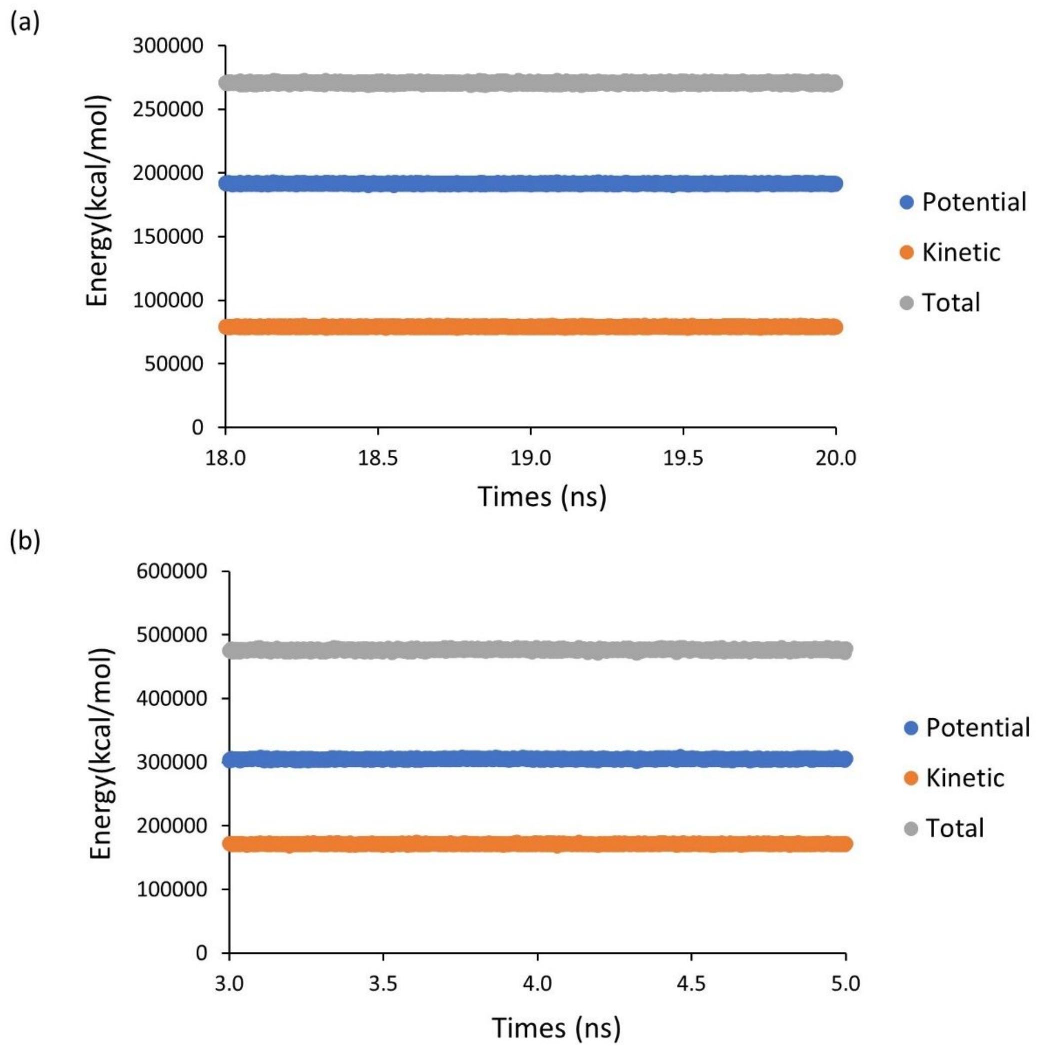

Figure S1. Energy profile of a random model with DS = 2.5 (R_ds2.5) over the last 2 ns of NPT run (a) in the last step (at 300 K, 1 bar) of 21-step decompression, (b) equilibration at 650 K, 1bar

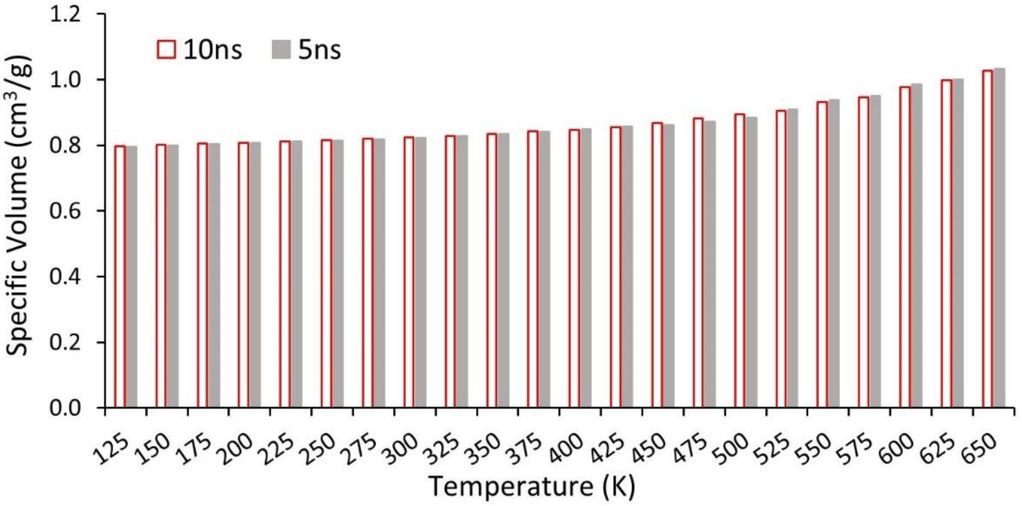

Figure S2. Comparison of specific volume between NPT run time 10 ns (red-white) and 5 ns (gray) during the cooling down process for U_O2 model (i.e., DS =1, substitution at O2).

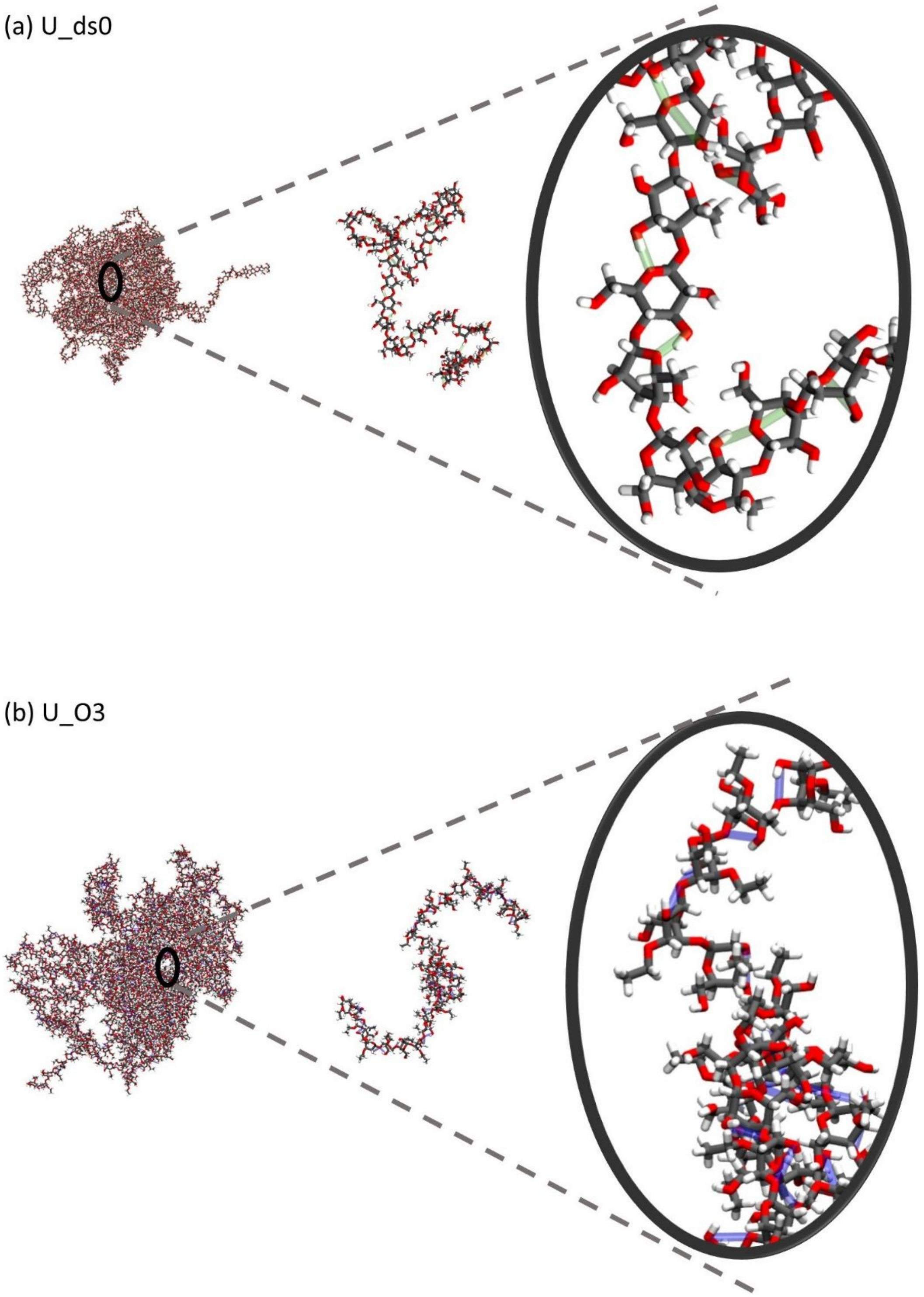

Figure S3 (a) The U_ds0 system where pairs of O3-O5 having a distance that is less than 3.5 Å are connected by lines in lime color, (b) U_O3 systems where pairs of O2-O4 having a distance that is less than 3.5 Å are connected by lines in violet color.

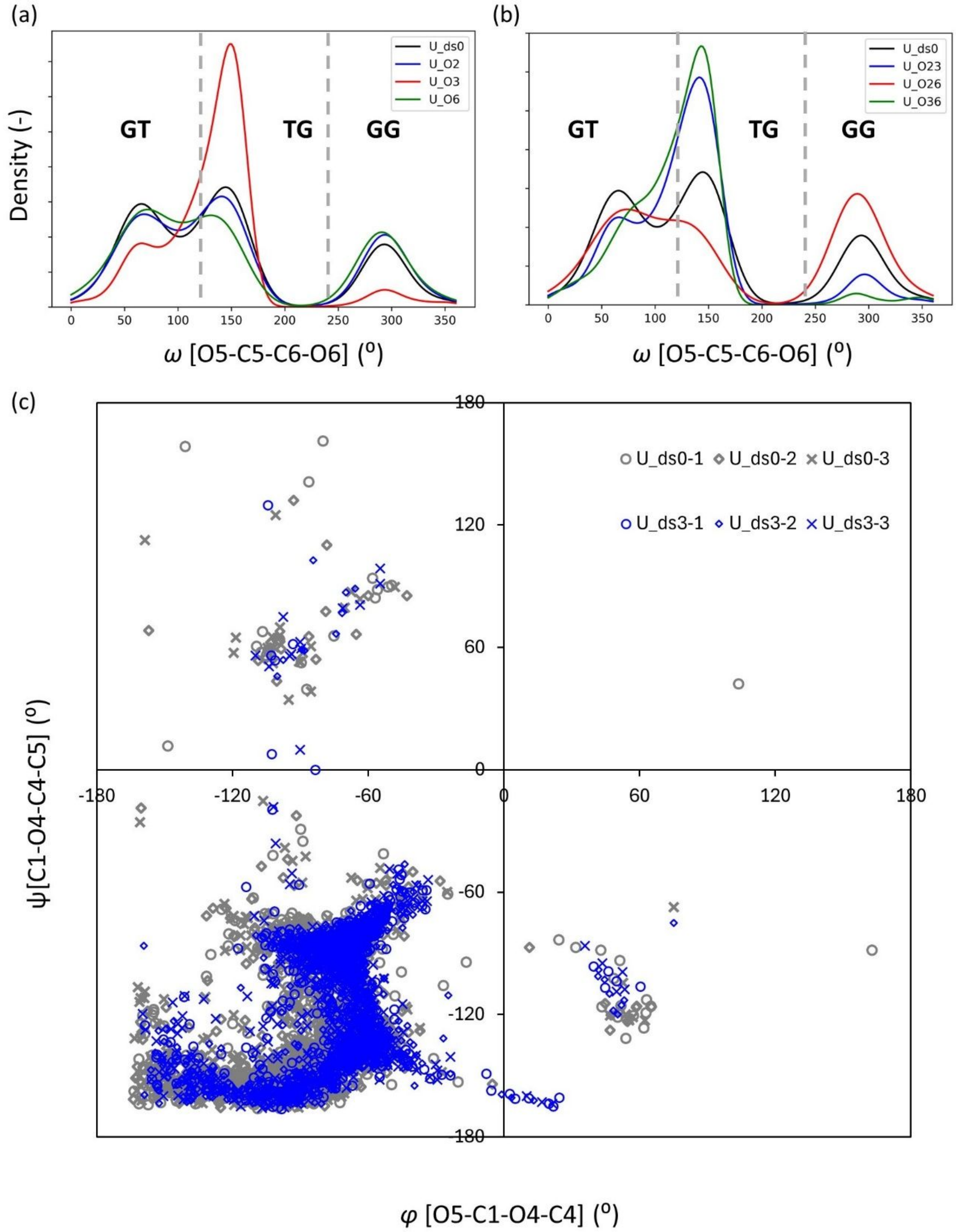

Figure S4. The distribution of the dihedral angle (ω: O5-C5-C6-O6) of (a) uniform models with DS = 1, (b) uniform models with DS = 2, (c) φ vs ψ plot of U_ds0 and U_ds3 models in the gray and blue colors, respectively.

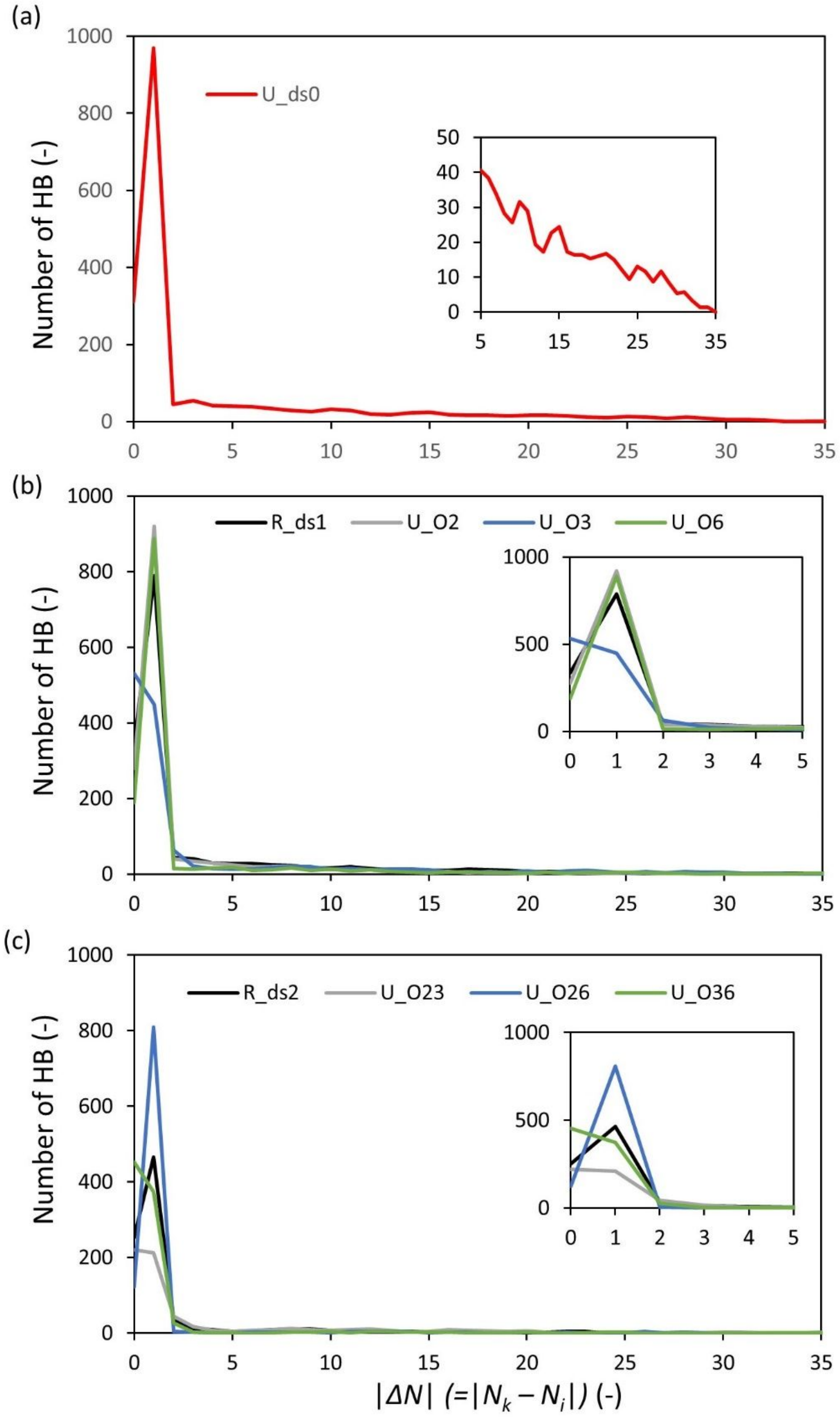

Figure S5. The number of the intramolecular hydrogen bond as a function of the HB length in the unit of the index of glucose units (see the Figure 1(a) about the way numbering the index of glucose units in a chain). (a) U_ds0, (b) U_O2, U_O3, U_O6 compared to U_ds0, (c) U_O23, U_O26, U_O36 compared to U_ds0, where the insets are the zoomed in figures up to a distance of 5-glucose-units.

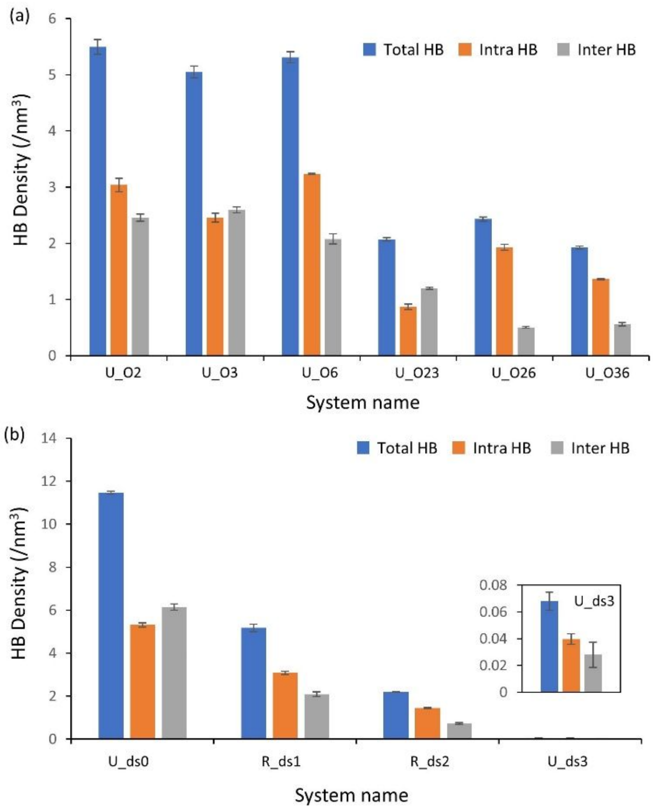

Figure S6. Hydrogen bond density of (a) uniform models with DS = 1 and 2 which can have three different models depending on the location of substituents, random models (b) with DS = 0 ,1, 2, and 3. Note that U_ds0 and U_ds3 in (b) are uniform models since the former is not substituted at all, and the latter is fully substituted. Total, Intramolecular, and Intermolecular density are presented in color blue, orange, and gray, respectively.

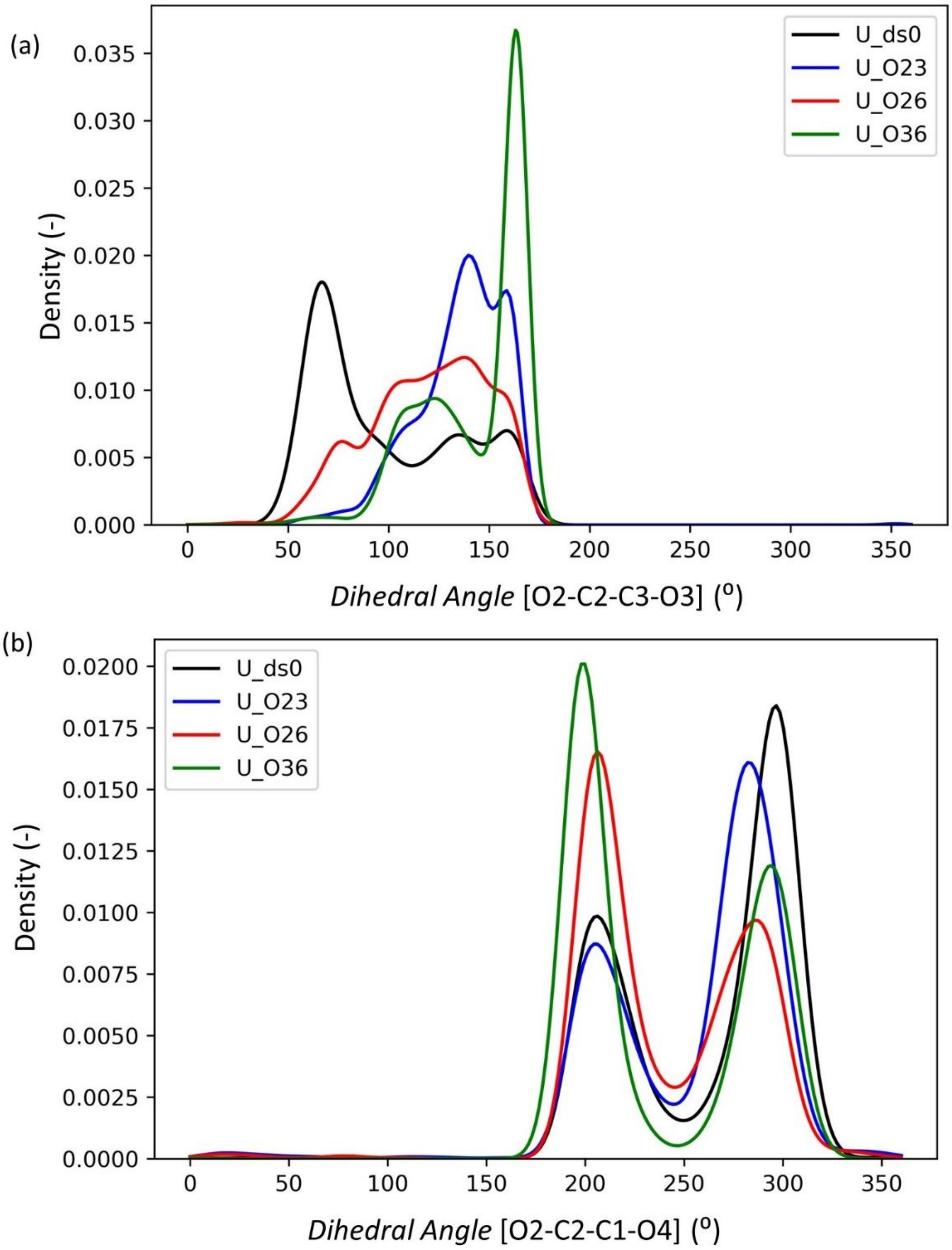

Figure S7. The distribution of dihedral angle (a) O2-C2-C3-O3 (b) O2-C2-C1-O4 for uniform models with DS = 2.

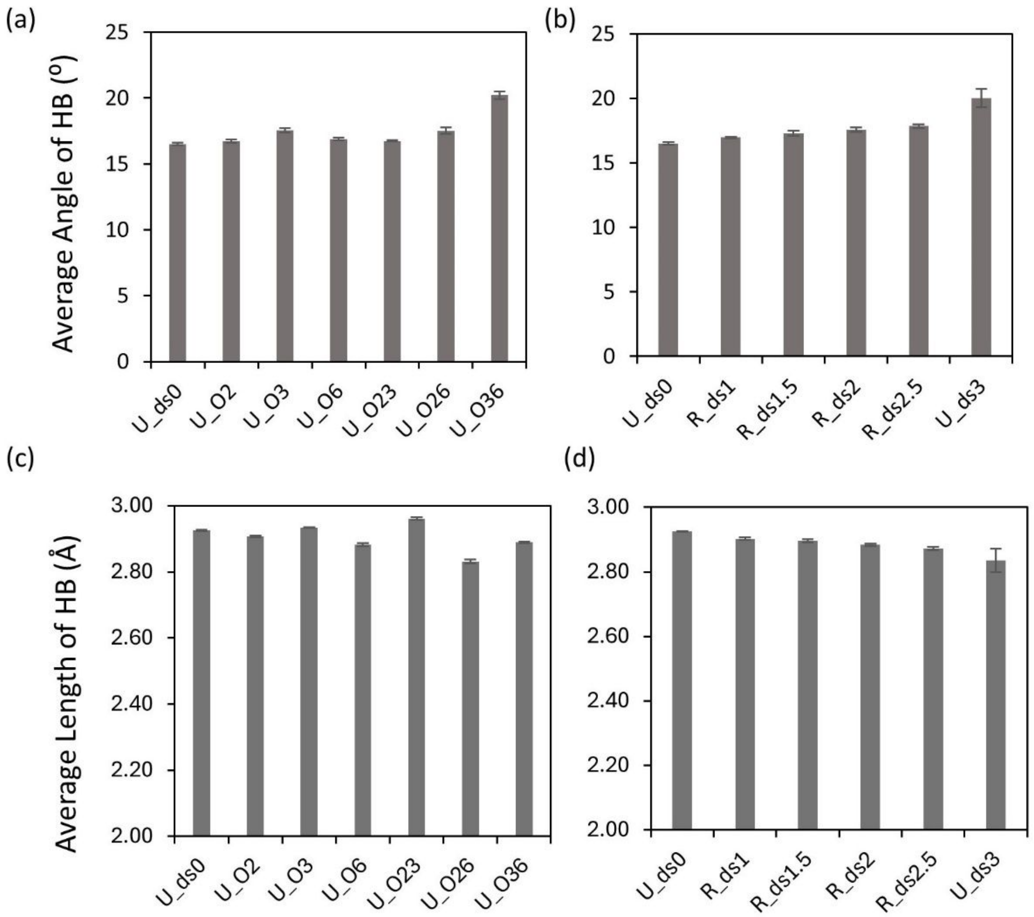

Figure S8. Average angle and length of hydrogen bonds: angle (a) uniform models, (b) random models, length (c) uniform models, (d) random models.

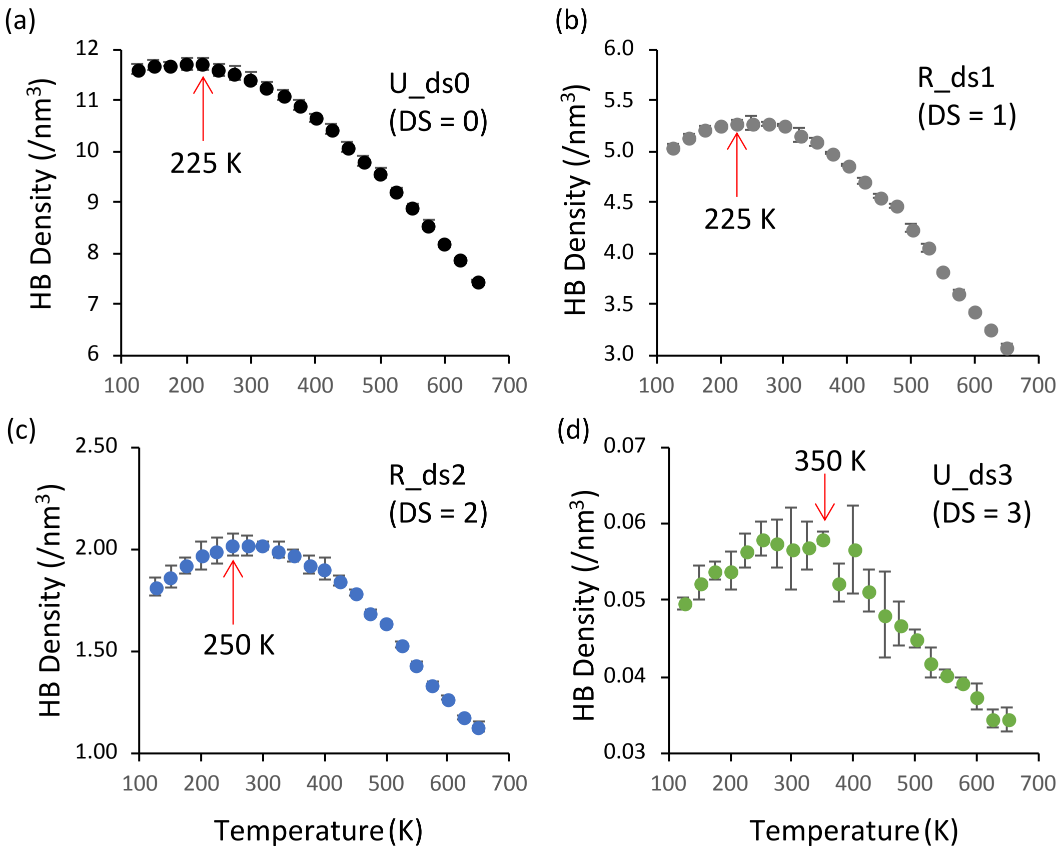

Figure S9. A change of HB density of (a) U_ds0 (DS = 0), (b) R_ds1 (DS = 1), (c), R_ds2 (DS = 2), and (d) U_ds3 (DS = 3). The points and error bar of plots are the average value and the standard deviation of three samples.

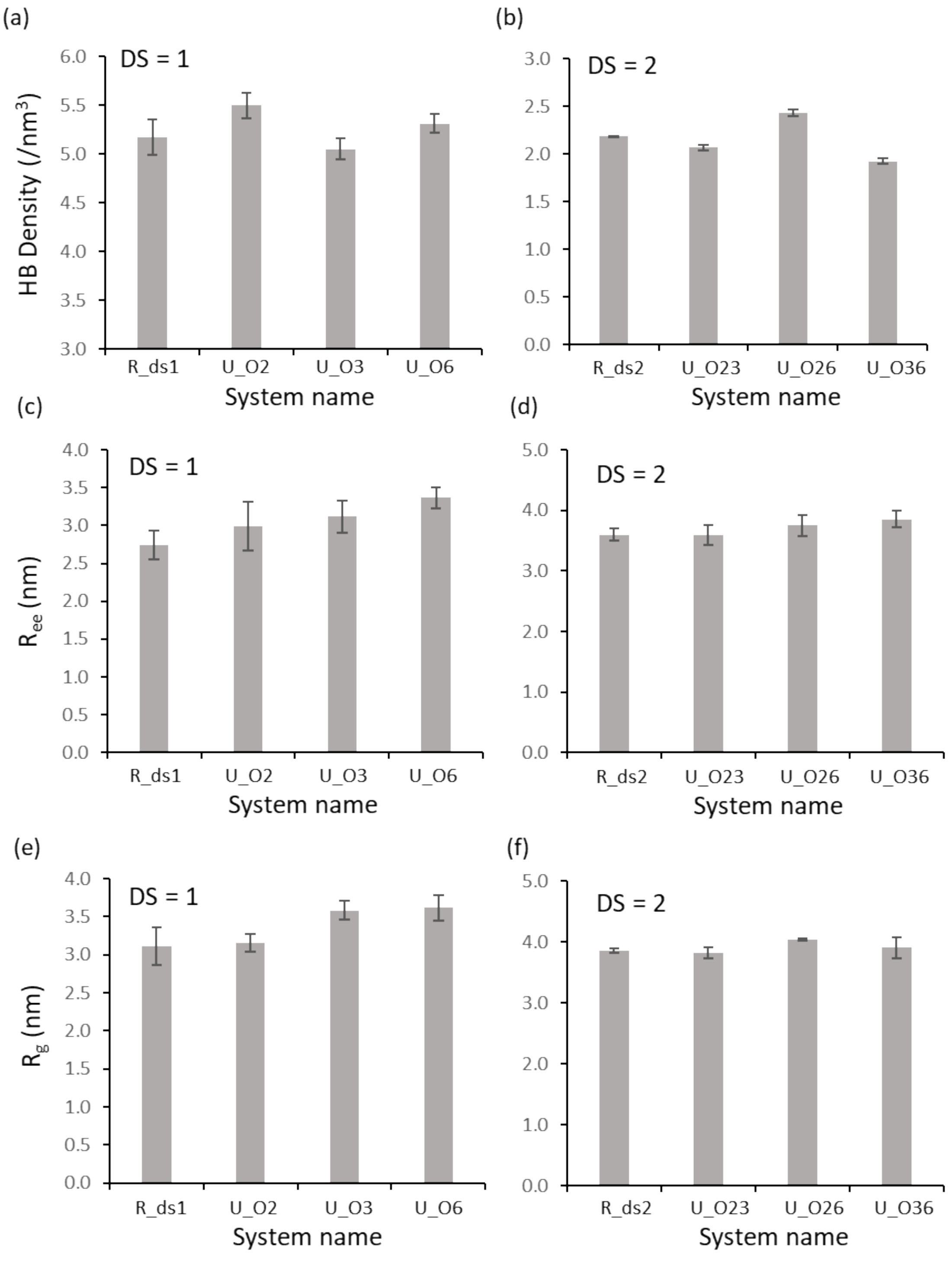

Figure S10. The comparison between models with DS = 1 and 2 in terms of (a) HB density, (b) end-to-end distance (R_{ee}), and (c) radius of gyration (R_g).

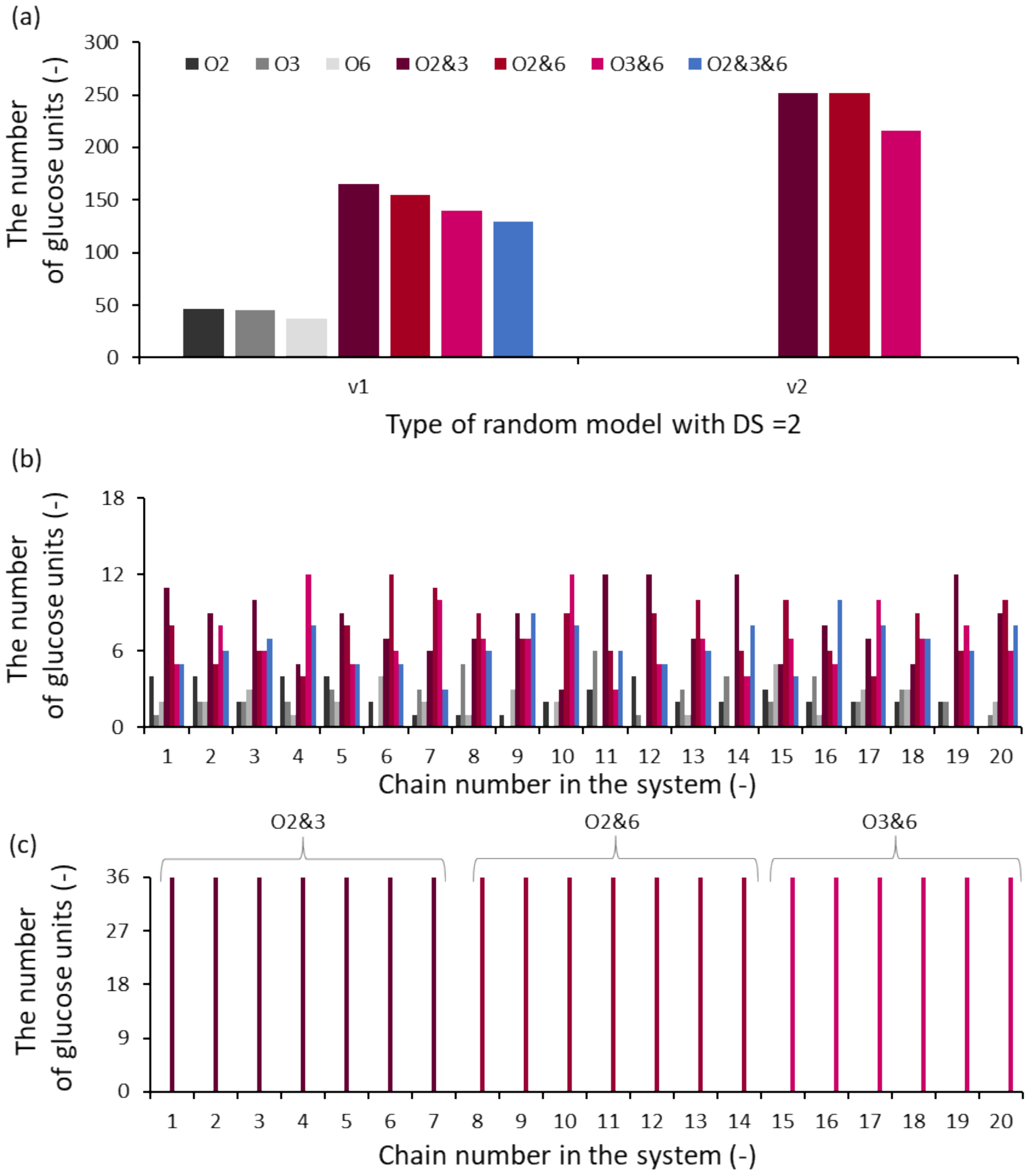

Figure S11. (a) The total number of substitution type of glucose units (O2, O3, O6, O2&3, O2&6, and O3&6 and O2&3&6) for random models R_ds2(v1) and R_ds2(v2), The number of substitution type of glucose units in terms of chains for (b) R_ds2(v1) and (c) R_ds2(v1). Since there is no anhydroglucose unit that is not substituted (i.e., ds = 0), the corresponding column is omitted. The sum of columns corresponding to a set of substitution type is 720 and 36 in (a) and (b-c) respectively.

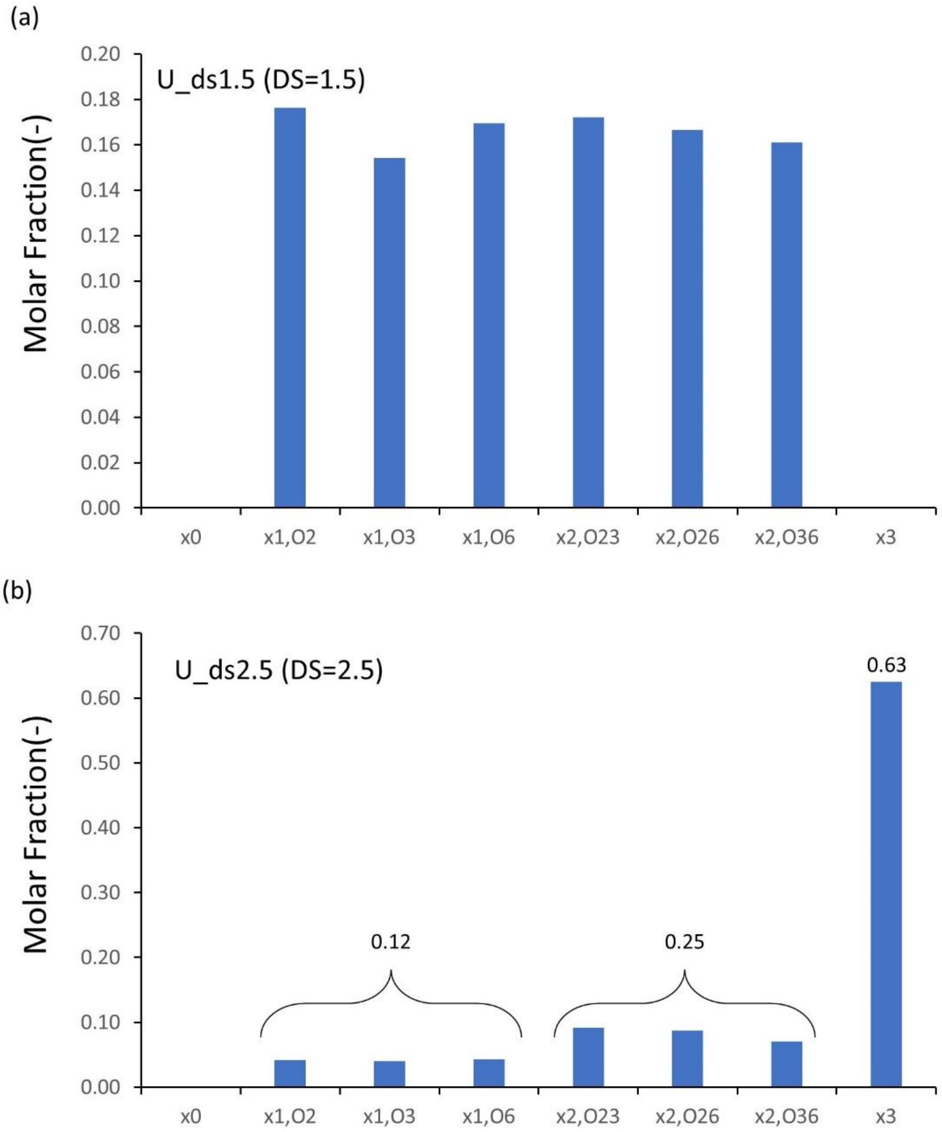

Figure S12. The molar fraction of each substitution type of glucose units in random models with DS = (a) 1.5 and (b) 2.5 for MD simulation.

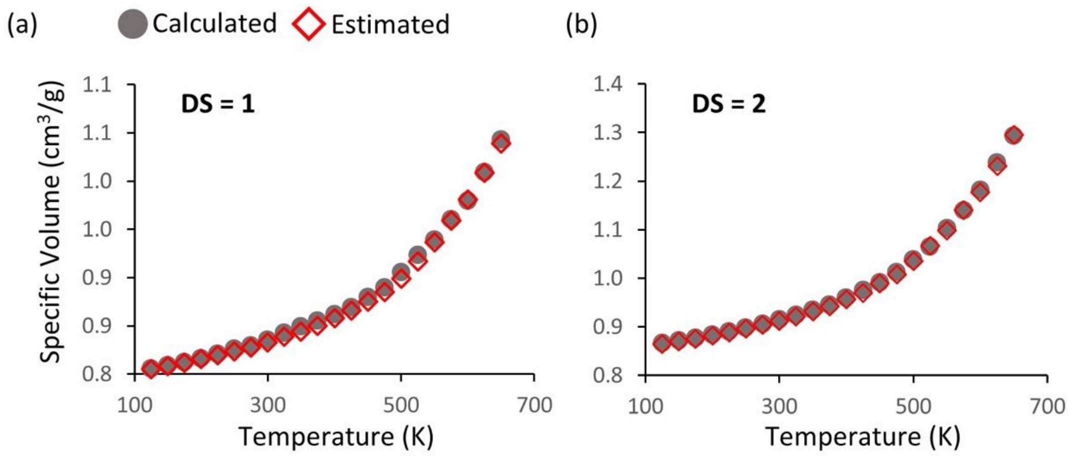

Figure S13. The PVT curves random models with a DS = 1 (a) and 2 (b) resulting from the MD simulation and prediction by Eq. 2. The MD results and predicted values are indicated by gray solid circles and red empty diamonds, respectively.

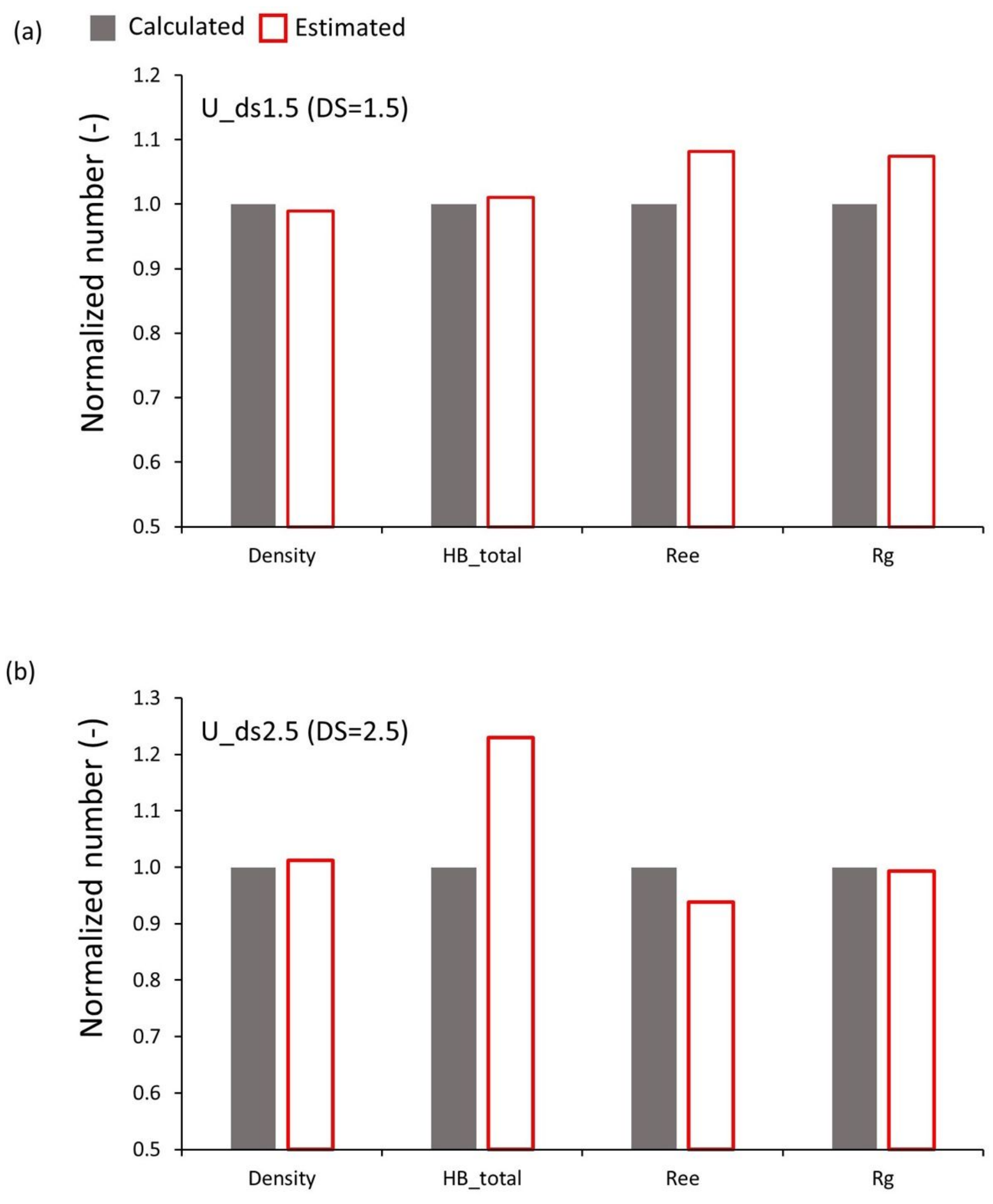

Figure S14. Comparison between MD simulation results and estimated values of random models with DS = 1.5 and 2.5 in terms of Density, HB density, end-to-end distance (R_{ee}), and radius of gyration (R_g) normalized by the value of MD results. The Normalized Value was calculated as Estimated value/Simulated value.

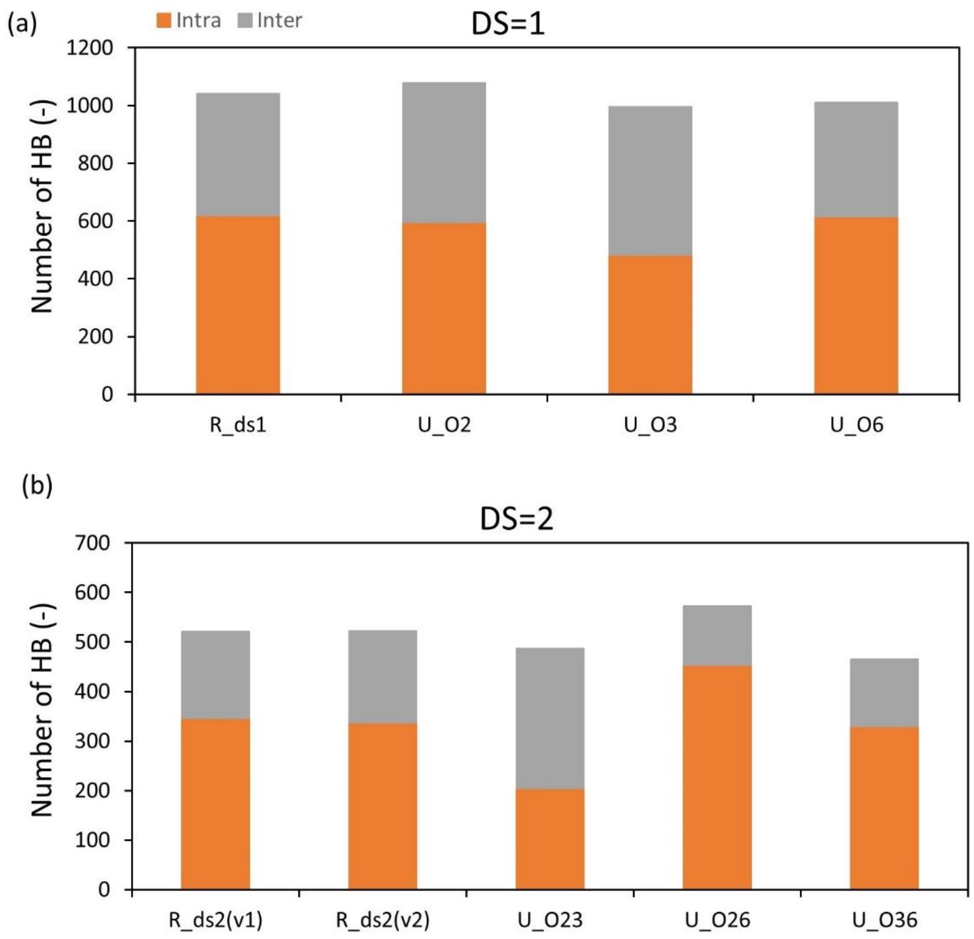

Figure S15. Comparison of total number of HB between models with the same DS (a) DS=1, (b) DS=2 where the orange and gray columns denote the intra- and inter-molecular HB.
